# Chemistry-Informed Machine Learning for Polymer Electrolyte Discovery

**DOI:** 10.1021/acscentsci.2c01123

**Published:** 2023-01-23

**Authors:** Gabriel Bradford, Jeffrey Lopez, Jurgis Ruza, Michael A. Stolberg, Richard Osterude, Jeremiah A. Johnson, Rafael Gomez-Bombarelli, Yang Shao-Horn

**Affiliations:** † Department of Mechanical Engineering, Massachusetts Institute of Technology, 77 Massachusetts Avenue, Cambridge, Massachusetts02139, United States; ‡ Research Laboratory of Electronics, Massachusetts Institute of Technology, 77 Massachusetts Avenue, Cambridge, Massachusetts02139, United States; § Department of Chemistry, Massachusetts Institute of Technology, 77 Massachusetts Avenue, Cambridge, Massachusetts02139, United States; ∥ Department of Materials Science and Engineering, Massachusetts Institute of Technology, 77 Massachusetts Avenue, Cambridge, Massachusetts02139, United States

## Abstract

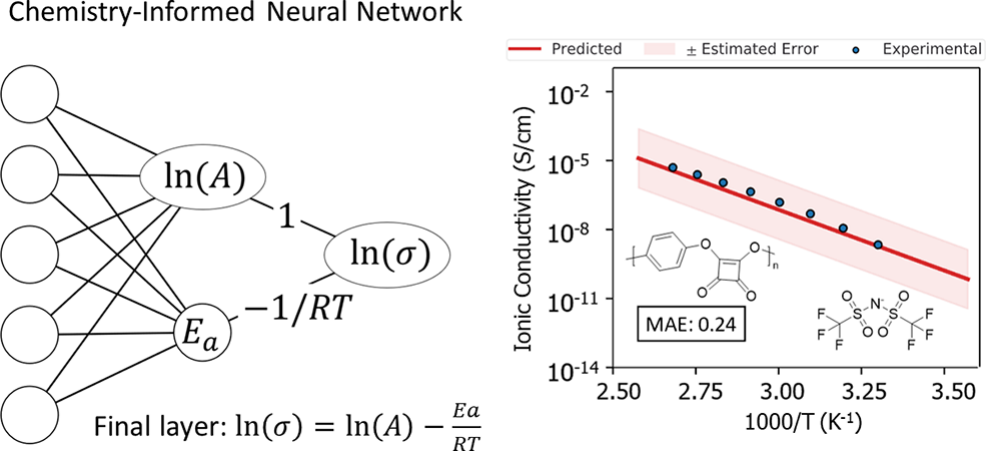

Solid polymer electrolytes (SPEs) have the potential to improve lithium-ion batteries by enhancing safety and enabling higher energy densities. However, SPEs suffer from significantly lower ionic conductivity than liquid and solid ceramic electrolytes, limiting their adoption in functional batteries. To facilitate more rapid discovery of high ionic conductivity SPEs, we developed a chemistry-informed machine learning model that accurately predicts ionic conductivity of SPEs. The model was trained on SPE ionic conductivity data from hundreds of experimental publications. Our chemistry-informed model encodes the Arrhenius equation, which describes temperature activated processes, into the readout layer of a state-of-the-art message passing neural network and has significantly improved accuracy over models that do not encode temperature dependence. Chemically informed readout layers are compatible with deep learning for other property prediction tasks and are especially useful where limited training data are available. Using the trained model, ionic conductivity values were predicted for several thousand candidate SPE formulations, allowing us to identify promising candidate SPEs. We also generated predictions for several different anions in poly(ethylene oxide) and poly(trimethylene carbonate), demonstrating the utility of our model in identifying descriptors for SPE ionic conductivity.

## Abstract 2

A machine learning model incorporating the Arrhenius relationship is trained on a large experimental data set to improve ionic conductivity predictions in polymer electrolytes.

## Introduction

Solid polymer electrolytes (SPEs) have been studied for decades as potential replacements for liquid organic electrolytes commonly used in batteries.^1−3^ SPEs can be engineered to offer several advantages over liquid electrolytes such as improved electrochemical stability^4^ and decreased flammability,^5^ advantages that are becoming more important as the demand for energy storage rapidly increases.^6^ However, compared to conventional organic liquid electrolytes, SPEs suffer from low ionic conductivity, limiting their use in practical devices.^7,8^ For application in practical lithium-ion batteries, electrolyte ionic conductivity must be at least 10^–3^ S/cm at room temperature,^9,10^ whereas state-of-the-art polymer electrolytes have only reached on the order of 10^–4^ S/cm at room temperature.^11−13^ While experimental and computation efforts have yielded improved ionic conductivity and better understanding of SPE systems,^14^ the cost in time and materials of experimental characterization along with the complex nature of novel polymer development have limited progress toward functional SPEs.^9,15^

In recent years, machine learning (ML) has been integrated into materials design workflows as a complement to experiments and simulations to accelerate discovery of a wide range of materials,^16−18^ including lithium-ion batteries.^19^ ML models can provide inexpensive and accurate materials property predictions which can be used to guide experimental efforts toward materials likely to meet desired design criteria.^20^ While the complexity of polymer systems can pose challenges to ML model development, recent work has leveraged ML to advance materials discovery for polymer separation membranes,^21^ polymer solar cells,^22^ thermally conductive polymers,^23,24^ and polymer dielectrics.^25^ Polymer electrolytes are no exception, with several works applying ML to improve or analyze molecular dynamics (MD) simulations on polymer electrolytes.^26−31^ For example, Xie et al. developed an ML model that corrected properties calculated from unconverged 5 ns MD simulations of SPEs to give accuracy similar to properties calculated from converged simulations, demonstrating the ability of ML to enhance and accelerate MD property screening.^28^ In addition to application of ML to MD simulation workflows, a few works have developed machine learning models trained on experimental data,^32,33^ although data availability often limits progress. In one work, an ML model was trained to predict ionic conductivity in a PEO-LiPF_6_ electrolyte while varying salt, plasticizer, and filler concentrations.^32^ The model predicted well for the specific PEO-LiPF_6_ system but was not able to generalize to other systems, due to a lack of diverse training data. In another work, Hatakeyama-Sato et al. developed an ML model trained on manually collected experimental data to predict ionic conductivity in doped glassy lithium conducting polymers, although the model’s ability to generalize to other types of polymer electrolytes was not reported, again likely in part due to the difficulty of gathering training data.^33^ While efforts to automate extraction of experimental materials properties data sets with ML are progressing,^34−36^ these efforts have not yet been applied to SPEs.

Beyond issues of data scarcity, many previous works attempting to predict materials properties of SPEs rely on models that are trained on molecular fingerprints^37−40^ to predict certain properties. Recently, models have been developed that predict materials properties using molecular structures directly as inputs.^41−43^ These models are differentiable end to end, which allows them to learn optimal weights to featurize molecules for specific prediction tasks without relying on fingerprinting methods that may lose relevant information. While traditional fingerprinting schemes have proven useful for certain property prediction tasks,^21,23−25^ Yang et al. showed that, given sufficient training data, learned representations outperformed traditional fingerprints across a variety of prediction tasks.^44^ Another useful development has been the incorporation of known physical or chemical constraints into ML models, which has been shown to improve accuracy and generalizability of model predictions.^45−47^ For example, Karpatne et al. developed a physics-informed ML model to model lake water temperature.^45^ They showed that an ML model trained with the constraint that water density must increase monotonically with depth outperformed the same ML model with the constraint removed.

In this work we build on recent advances in machine learning to develop a chemistry-informed ML model that predicts SPE ionic conductivity based on the electrolyte molecular structure and composition alone. To train our model, we gathered the largest known data set of SPE ionic conductivity values from 217 experimental publications. Our model leverages a fully differentiable message passing architecture^44^ to learn optimal representations of the molecular components of SPEs coupled to a chemically informed Arrhenius regularization built into the model. We used our model to screen over 20,000 potential SPEs composed of various commonly used lithium salts with synthetically accessible polymers^48^ which had not previously been evaluated as SPEs, identifying promising polymers for future experimental characterization. We also screened several different lithium salts with two polymers for which ample training data were available, ensuring accurate predictions from the model. From these predictions, we analyzed the effect of different anion descriptors on ionic conductivity and found that changing the polymer structure leads to reversed correlations between predicted ionic conductivity and anion descriptors such as molecular weight or interaction strength.

## Methods

### Experimental Data Set Extraction

A training set of experimental measurements of SPE ionic conductivity was extracted from three sources. The first source was a corpus of 135 publications curated from existing SPE literature from which we extracted 7,133 ionic conductivity data points. Each publication was screened for indications of rigorous experimental practice according to criteria outlined in Supporting Information (SI) Table S1. Ionic conductivity values were extracted manually from selected publications, along with associated electrolyte formulation information, which consisted of polymer structure, polymer molecular weight, salt structure, and salt concentration. Additional data, such as glass transition temperature, dispersity, and type and concentration of ceramic or organic additives, were also extracted where available. In addition to the data manually collected, we extracted data from two previously published data sets,^33,49^ from which we obtained 6,250 data points. We checked the publications cited in each data set and extracted data from publications meeting the criteria in Table S1.

### Machine Learning Model Development

**Figure 1:**
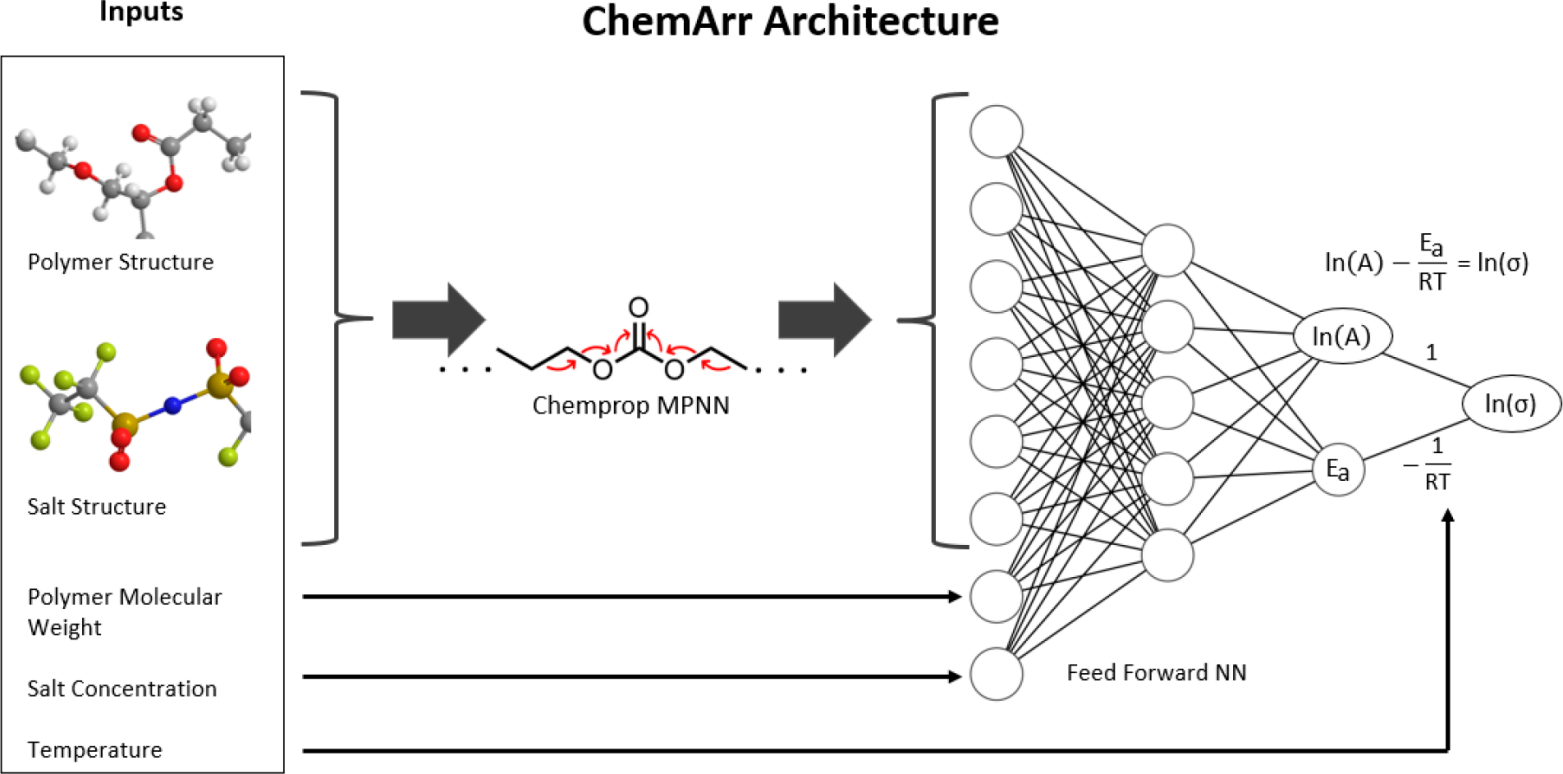
Diagram of ChemArr model architecture. The model uses the message passing neural network (MPNN) from Yang et al.^44^ to featurize the polymer and salt structures, while numeric features are concatenated to the molecular feature vector resulting from the MPNN. The penultimate layer of the model outputs the parameters of the Arrhenius equation, which are then passed through the Arrhenius equation with the input temperature to generate an ionic conductivity prediction.

A machine learning model, ChemArr, was developed using the message passing neural network (MPNN) architecture of the Chemprop model released by Yang et al.^44^ The entire MPNN is differentiable, allowing message passing layers to learn optimal weights to transform molecular graphs into numerical vectors. The ChemArr model utilizes five input features: (1) the polymer structure as a molecular graph, (2) the salt structure as a molecular graph, (3) the logarithm of the polymer molecular weight, (4) the salt concentration in units of moles of salt per kilogram of polymer, and (5) temperature. Each polymer was represented as an oligomer with at least 50 heavy atoms capped by methyl end groups. ChemArr uses Chemprop’s original message passing architecture, which was used to generate numeric feature vectors from the polymer and salt molecular graphs in each SPE. The oligomer and salt molecular graphs were input together as a disconnected graph, allowing the MPNN to learn the representation of polymer and salt together. Following featurization by the MPNN, the salt concentration and polymer molecular weight values were concatenated to the numeric molecular feature vector which was then passed through a feedforward neural network. The final layers of the feedforward network were modified such that the penultimate layer outputs the parameters ln (*A*) and *E*_a_ of the Arrhenius equation for ionic conductivity, which in the logarithmic linearized form is
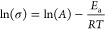
1where σ is ionic conductivity, *A* is the prefactor, *E*_a_ is activation energy, *R* is the ideal gas constant, and *T* is temperature. (The logarithmic linearization of the Arrhenius equation was used since the values of *A* and σ span several orders of magnitude.) The final layer of the model has fixed weights that replicate the Arrhenius equation to calculate ln(σ) from model outputs ln(*A*) and *E*_a_, as well as the temperature *T* of the input. Figure 1 shows a diagram of the model. ChemArr was trained using a mean squared error loss function.

To estimate the error for predictions of individual electrolyte formulations, we implemented a modified version of a distance-based error approach described by Liu et al.,^50^ in which error is estimated as a function of the chemical distance between a single predicted data point and the training data (see the SI for details).

### Machine Learning Model Benchmarks

ChemArr was benchmarked against Yang et al.’s Chemprop model and XGBoost,^51^ a gradient boosted decision tree algorithm, both of which were trained to predict ionic conductivity without incorporating the Arrhenius equation. The Chemprop model used the same input structure as ChemArr, except that temperature was concatenated to the MPNN feature vector with the other numerical inputs rather than being used in the final Arrhenius layer. In the XGBoost model, the polymer and salt for each electrolyte were represented with 256-bit Morgan fingerprints^40^ which were concatenated with polymer molecular weight, salt concentration, and temperature.

During benchmarking, 10-fold cross-validation was performed. In each fold, the data were split into separate train, validation, and test splits, where each test and validation set contained only polymers that did not appear in the training set. To select the test and validation data for each fold, we selected 10% of the polymer structures for the test set and 20% of the structures for the validation set. Since our data sets contain a different number of data points for each polymer structure, we selected combinations of structures such that the test and validation sets contained as close to 10% and 20% of the total data, respectively, as possible. In each cross-validation fold, five independent models with different random initializations were trained on data from the remaining 70% of the polymer structures and error statistics were calculated using the average of the five models. The process was repeated 10 times so that each polymer appeared in a test set only once. The only exception was that polyethylene-oxide (PEO) containing data were excluded from all test sets to assess the ability of the model to predict non-PEO SPEs, for which experimental data are sparsely available.

Model performance was assessed with two metrics, mean absolute error and the Spearman rank correlation coefficient, or Spearman R. Mean absolute error averages the absolute error of all test set predictions, giving a general picture of how close model predictions are to the ground truth values. Spearman R measures how well the model ranks different values, regardless of how close the model predictions are to the ground truth.^52^ Spearman R ranges from −1, indicating an exact reverse rank order, to 1, indicating exactly matched rank orders. Although interpretations vary, values at or above 0.6 are considered to indicate a strong rank correlation.^53^

### Novel Electrolyte Screening

An exploration of potential novel polymer electrolytes was conducted by first collecting a screening set of 820 polymers from PolyInfo,^48^ a database containing experimental data for over 13,000 synthetically available polymers. Candidate polymer electrolyte formulations were generated by combining polymers from the screening set with 10 commonly used lithium salts at 3 different salt concentrations, yielding over 20,000 unique formulations for screening. Ionic conductivity predictions and error estimates for the screening set were made with ChemArr trained on the entire experimental data set. Screening was also done for 20 lithium salts with different anions in PEO and poly(trimethylene carbonate) (PTMC) to investigate how ionic conductivity is affected by the anion in the electrolyte and how anion effects differ in different polymers. In each polymer, formulations were screened at 1.5 mol of salt per kg of polymer at a temperature of 25 °C.

## Results and Discussion

### Polymer Electrolyte Ionic Conductivity Data Set

**Figure 2:**
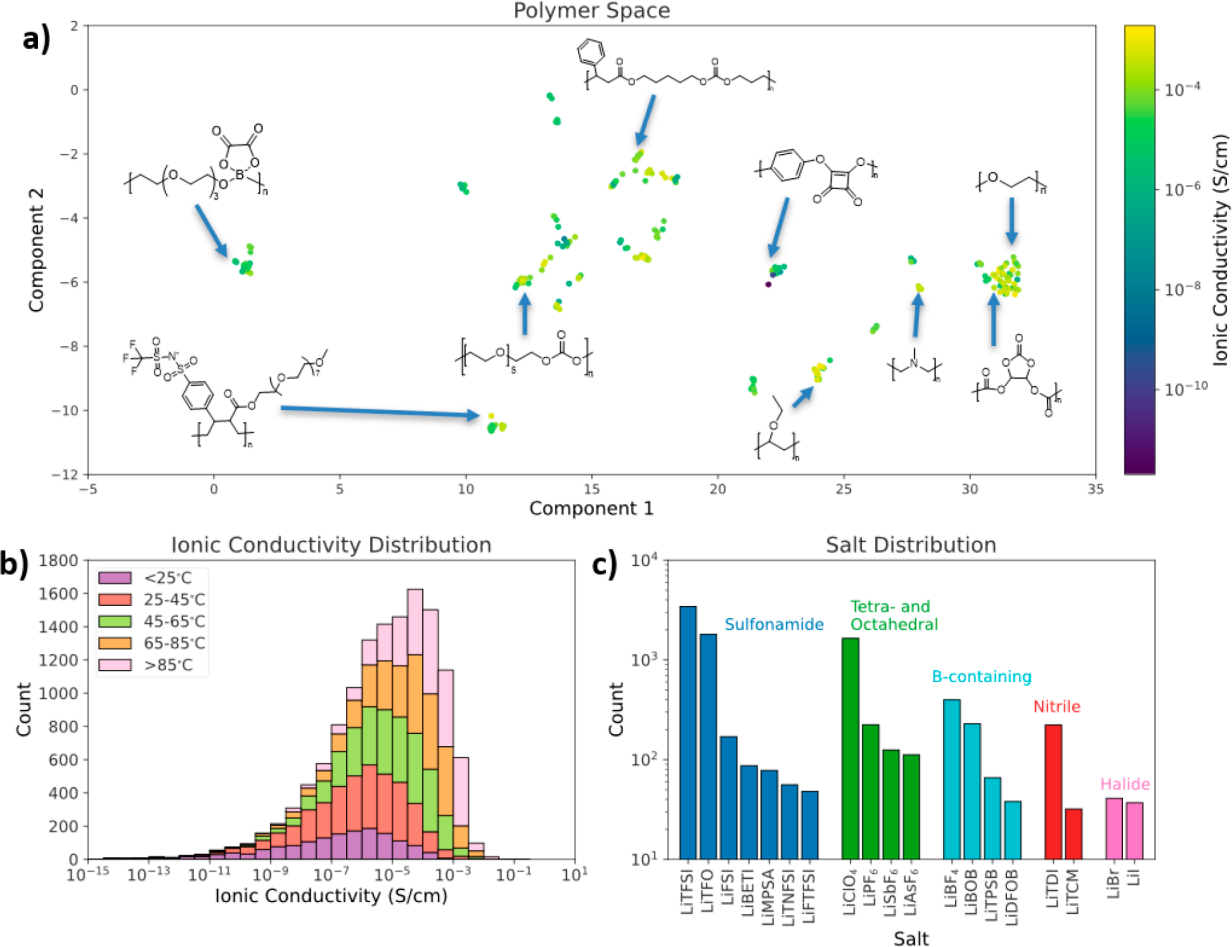
(a) Representation of the polymer space of the experimental data set. The *x* and *y* axes show the principal components of the polymer structures, which were generated using UMAP,^54^ a dimensionality reduction technique, on 128-bit Morgan fingerprints of the polymer structures. Several structures are labeled to illustrate the diversity of the training data. Points are colored according to experimental ionic conductivity at 80 °C. (b) Distribution of ionic conductivity values for all data in the experimental data set. Data are separated into temperature ranges. (c) Distribution of the 20 most prevalent salts in the experimental data set grouped by chemically similar anions.

Figure 2a shows a projection of the polymer space covered by our experimental data set. The projection provides a qualitative visualization of our data set, allowing us to view regions that are densely and sparsely populated, indicating structural motifs that are more or less common in our experimental data set. The large cluster of points in the right area of Figure 2a captures the PEO-like polymers in the data set. Much experimental research has focused on PEO-like polymers, and the composition of our training data reflects that. Outside of PEO-like polymers, the projection shows a broad coverage of diverse polymers, albeit sparsely populated in many areas. In areas with few or no training data, we expect model predictions to be less accurate. Future experimental work on diverse polymers that fills the gaps in our training data would allow us to generalize model predictions to more exotic chemistries and develop more generalizable guidelines for polymer electrolyte development. Figure 2b shows the distribution of ionic conductivity values in our experimental data set at various temperature ranges. Few polymer electrolytes in our data set meet the target ionic conductivity of 10^–3^ S/cm—and only do so at elevated temperatures. Most room-temperature data fall in the range of 10^–9^ to 10^–4^ S/cm. Figure 2c shows the distribution of the 20 most common salts in the data set. Three salts—lithium bis(trifluoromethanesulfonyl)imide (LiTFSI), lithium trifluoromethanesulfonate (LiTFO), and lithium perchlorate (LiClO_4_)—cover over one-third of the data, reflecting their popularity in the field. However, several other salts are present in significant proportions, with 10 having over 100 data points. Table 1 lists summary statistics for the training data. The data set contains over 12,000 ionic conductivity data points for over 3,000 unique electrolyte formulations, where a unique electrolyte is defined by the polymer, salt, salt concentration, polymer molecular weight, and additives if present. In addition to SPEs composed of uncharged polymers mixed with lithium salts, our data set contains ionic conductivity measurements for 90 different single-ion conductors, species with anions covalently tethered to a polymeric backbone that have shown promise for solid state electrolyte applications.^55−58^

**Table 1:** Summary Statistics of Experimental Data Set

Material Type	No. of Data Points	No. of Unique Species
Polymer	12,383	247
Salt	10,272	81
Single-ion conductor	2,111	90
		
Total	12,383	3,195

### Machine Learning Model Performance and Benchmarking

**Figure 3:**
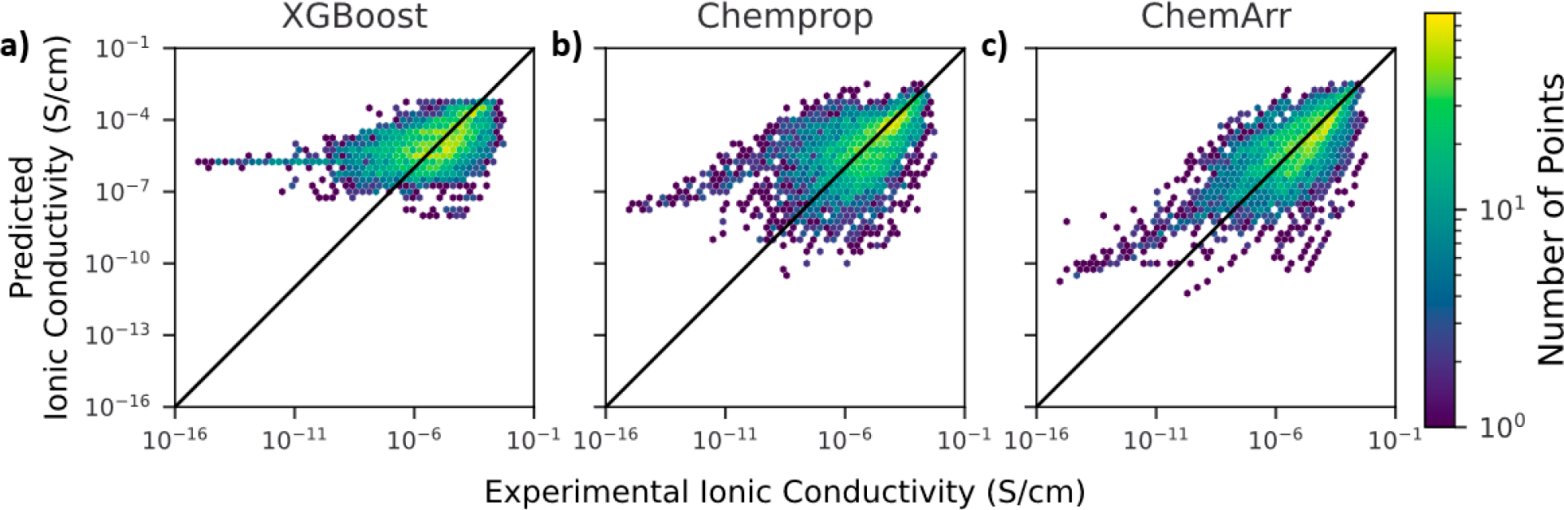
Predicted vs experimental ionic conductivity on cross-validation (see Methods) for (a) XGBoost, (b) Chemprop, and (c) ChemArr.

ChemArr was benchmarked against two other models, XGBoost and Chemprop, as described in Methods. The 10-fold cross-validation test mean absolute error (MAE) and Spearman R for each of the models are shown in Table 2, where XGBoost performs the worst, with Chemprop offering a slight improvement and ChemArr performing best. ChemArr also outperforms XGBoost and Chemprop when only high conductivity (>10^–4^ S/cm) data points are considered (Table S2 and Figure S3). For comparison of ChemArr models trained with different molecular representation methods or with the DFT calculated interaction energy between the polymer and Li^+^ ion, see Tables S3 and S4. Since MAE was calculated using the log of conductivity, a value of 1 indicates an average error of 1 order of magnitude. For comparison, well-conducted experimental measurements of ionic conductivity in SPEs can have errors up to a half an order of magnitude or 0.5 log(*S*/cm) on the log scale between replicate measurements in the same study.^59,60^ ChemArr’s inclusion of the Arrhenius equation yields a 7% reduction in MAE over the unaltered Chemprop model. The 32% improvement in Spearman R shows that ChemArr comparatively ranks ionic conductivity of different SPE formulations more consistently with experimental data than either Chemprop or XGBoost. Improved ranking performance will be particularly valuable if the model is used to select novel SPE candidates for experimental characterization with limited time and resources. Panels a–c of Figure 3 show the predicted vs experimental ionic conductivity values for the three models tested. Notably, ChemArr’s improvement over the other models is most pronounced for low ionic conductivity values (below 10^–8^ S/cm), where ChemArr’s predictions are orders of magnitude closer to experimental values than those of the other models. ChemArr’s improved predictions result from the encoded Arrhenius temperature dependence. By leveraging explicit temperature dependence, patterns learned from high-temperature data, which are more common in the experimental literature, can be extended to low-temperature predictions, where most known SPEs exhibit too low conductivities. This ability to generalize is a valuable trait for screening SPEs to be used at room temperature, while still leveraging high-temperature training data.

**Table 2:** Performance Metrics for Each of the Models Tested

	Mean Absolute Error (log(*S*/cm))	Spearman R
XGBoost	1.09 + - 0.027	0.38 + - 0.026
Chemprop	1.08 + - 0.012	0.45+-0.006
ChemArr	**1.00 + - 0.030**	**0.59 + - 0.022**

**Figure 4:**
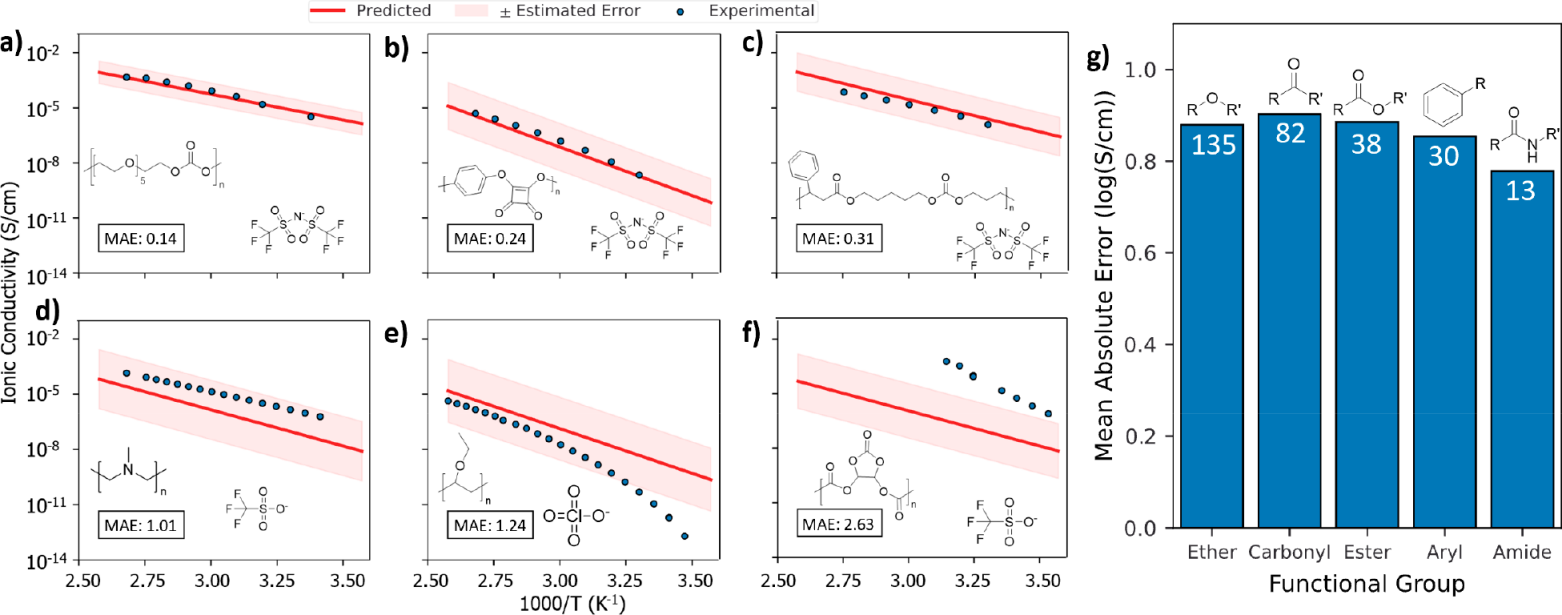
(a–f) Arrhenius plots of predicted and experimental ionic conductivity for six SPEs from the test set. The mean absolute error (MAE) (units of log(*S*/cm)), polymer, and salt for each formulation are inlaid on the plot. The estimated error for each prediction is shown in light red. The plots show predicted and experimental data for (a–c) high-accuracy predictions for various polymer types; (d) a formulation for which the MAE was 1, illustrating the upper error bound for 80% of predictions; (e) a formulation with strong non-Arrhenius behavior; and (f) a formulation with high prediction error for which the experimental data were later determined to be unreliable. (g) Mean absolute error for predictions made on all SPEs with polymers containing the listed functional group. The number of polymers containing each functional group is shown in white on each bar.

Panels a–f of Figure 4 show predicted and experimental ionic conductivities for 6 example SPEs drawn from cross-validation test sets, with the MAE and polymer and salt structures inlaid on each plot. Panels a–c of Figure 4 show Arrhenius plots of three different polymer electrolyte formulations for which predictions were well within the range of experimental error. Across the cross-validation test sets, model predictions were within a half-order of magnitude of the experimental values (or MAE < 0.5 log(*S*/cm)) for 49% of all polymer species, demonstrating that ChemArr predicts near experimental accuracy for just below half of the SPE data that we have collected. Figure 4d shows the predicted and experimental values for a polymer without oxygen, one of only 8 oxygen-free polymers in our data. In this case the model predicted ionic conductivity with an MAE of 1.01 log(*S*/cm) or about 1 order of magnitude error. Since over 95% of the polymers in our training data coordinate lithium ions with oxygen, the increased error for the prediction shown in Figure 4d relative to Figure 4a–c can be attributed to a lack of training data for polymers that coordinate lithium with elements other than oxygen. Across all of the test data, 81% of SPEs were predicted within 1 order of magnitude of the experimental data.

Panels e and f of Figures 4 show examples where predictions did not match experimental values. Figure 4e shows a polymer that exhibits non-Arrhenius behavior. ChemArr gives accurate predictions at the high-temperature range but fails to account for the non-Arrhenius curve in the data, yielding high error at low temperatures. To address this, we attempted to develop a model based on the Vogel–Fulcher–Tammann (VFT) equation, which describes SPEs that do not follow Arrhenius behavior. However, the VFT-based model failed to accurately learn non-Arrhenius behavior, defaulting to linear Arrhenius behavior for all predictions. (See Figure S4 for details.) Figure S5 shows another instance where ChemArr is unable to capture experimental trends. In this case, a PEO electrolyte exhibits two different activation energies above and below the melting point resulting in two distinct slopes. Figure 4f shows an SPE that follows Arrhenius behavior reasonably well, yet the prediction is still quite different than the experimental values. However, the polymer shown in Figure 4f^61^ is known to decompose in the presence of lithium salts,^62,63^ making experimental measurements of ionic conductivity highly unreliable for this polymer. In this case, the high prediction error signaled problematic experimental data.

Figure 4g shows the MAE for predictions made on polymers containing various functional groups. The MAE of predictions for each functional group is less than 1, indicating that the model gives accurate predictions across a range of polymer chemistries. The MAE of about 0.8 log(*S*/cm) for esters, aryls, and amides is especially notable, given that there are relatively few polymers that contain those functional groups in our training data. The low error for these functional groups gives confidence that the model can be applied beyond traditional ether-based SPEs.

### Model Predictions on New Electrolyte Systems

**Figure 5:**
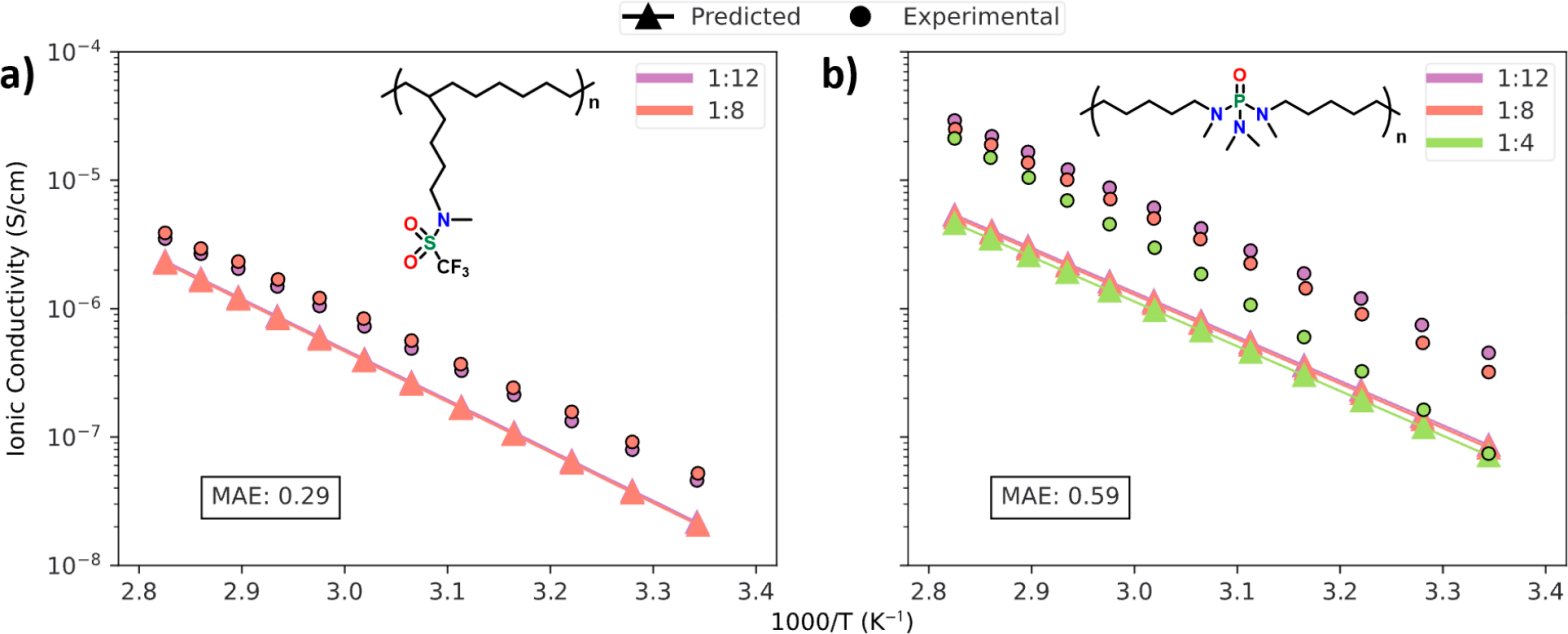
Model predictions and experimental values for two novel polymer electrolyte materials, consisting of LiTFSI mixed with (a) P_CODC_4_CF_3_SA and (b) P_C_10_PA_MC, two polymers which were developed and characterized in house with the chemical structure shown on the plots. The concentration in terms of lithium to monomer ratio for experimental and predicted values is shown in the legend. The mean absolute error (MAE) (units of log(*S*/cm)) of the predicted vs experimental values is inlaid in each plot.

ChemArr was also validated on experimental measurements for electrolytes based on two novel polymers, P_CODC_4_CF_3_SA and P_C_10_PA_MC, which were synthesized and characterized in-house as described by Feng.^64^ Panels a and b of Figure 5 show the predicted and experimental ionic conductivity values for both polymers mixed with LiTFSI. Figure 5a shows quite good agreement between the predicted and experimental values for P_CODC_4_CF_3_SA. The MAE of 0.29 log(*S*/cm) for the predictions is well within the general error of experiments. Figure 5b shows slightly poorer agreement between the predicted and experimental values than Figure 5a. However, with an MAE of 0.59 log(*S*/cm) for the data in Figure 5b, the model predictions are still in the same order of error as experiments. The model correctly predicts the activation energy (or slope) of the two lower concentration formulations but underestimates the prefactor (or intercept) of those formulations. The reason for the inaccurate activation energy for the 1:4 formulation is unclear, but it likely results from the novelty of the polymer to the machine learning model. The training data for this prediction model contained no examples of a phosphorus–oxygen double bond, which for the polymer shown in Figure 5b is likely the primary coordinating group. Given that the model has never seen this chemical moiety, it is perhaps unsurprising that the model is unable to fully capture how ionic conductivity changes as salt concentration increases. The model does, however, correctly predict the relative order of ionic conductivity for the three different salt concentrations, with the lowest concentration having the highest ionic conductivity and ionic conductivity decreasing with increasing salt concentration. The generally good agreement between ChemArr’s predictions and the experimental data gives confidence in the model’s ability to predict ionic conductivity for SPEs composed of novel polymers, even in the case, as in Figure 5b, where the primary lithium coordinating moiety is absent from the training data.

### Screening Novel Polymers for Ionic Conductivity

**Figure 6:**
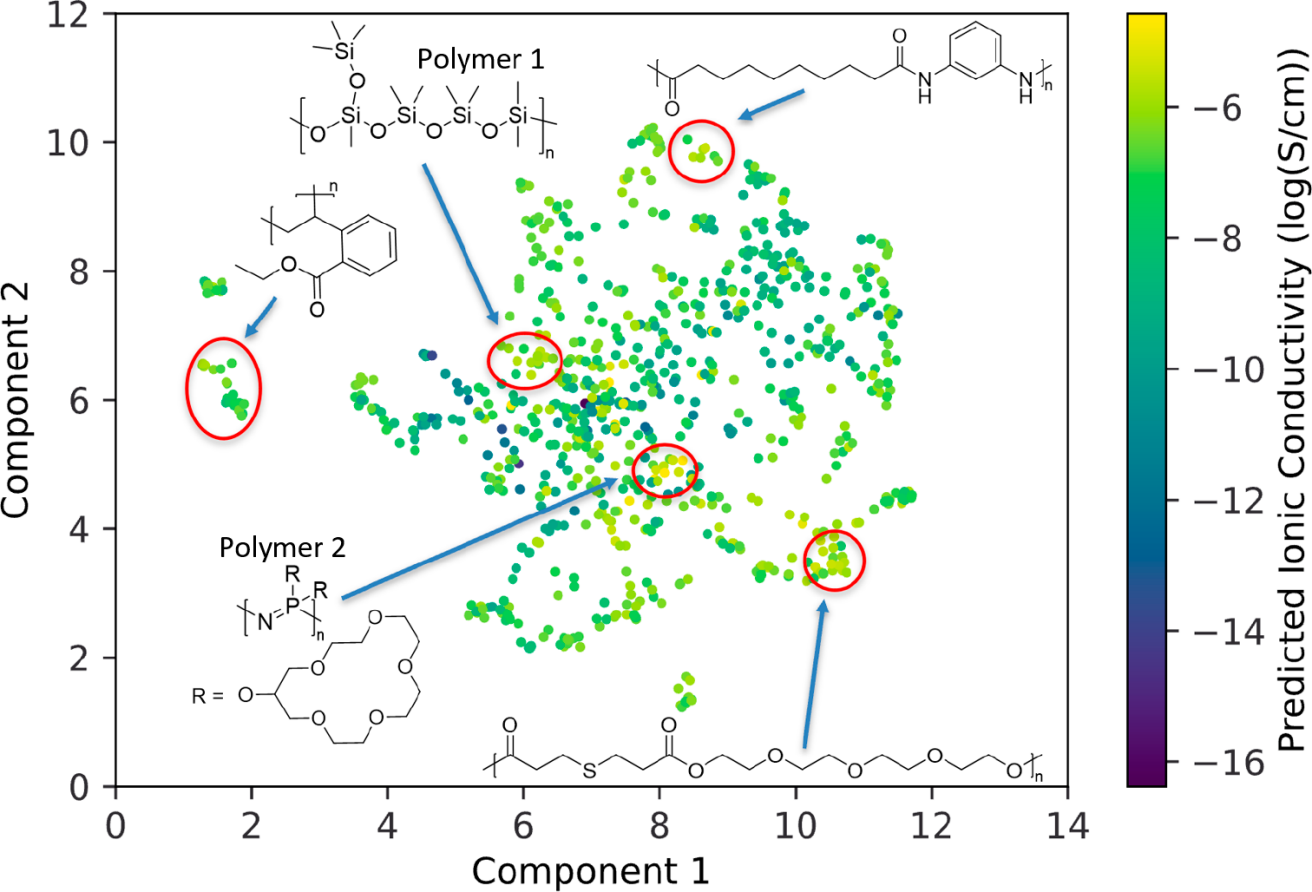
Representation of screened polymer space. Each point represents a different polymer with LiTFSI at 1.5 mol of salt per kg of polymer at 25 °C. Points colored according to predicted ionic conductivity. Several regions with high average predicted ionic conductivity are indicated, with a representative polymer structure labeling the region.

Following validation of ChemArr, the model was trained on all experimental data. We then generated ionic conductivity predictions for over 20,000 hypothetical SPE formulations derived from 820 synthetically available polymers as described in Methods. Figure 6 shows a two-dimensional projection of the space of predicted polymers, generated with UMAP,^54^ where similar polymer structures will be located close together. Visualizing the predicted ionic conductivities in this way allows us to identify groupings of highly predicted polymers, which can serve as a guide for experimental testing. Polymer 1 shows a highly predicted siloxane polymer structure. The high predictions for Polymer 1 and other similar structures are consistent with reports that siloxane polymers with low glass transition temperatures can have high ionic conductivity.^65,66^ Polymer 2 shows another interesting direction for exploration of new polymer electrolytes. Polymer 2 is structurally similar to poly(bis((methoxyethoxy)ethoxy)phosphazene) (MEEP), which has been characterized previously in SPEs and shows good ionic conductivity due in part to a low glass transition temperature enabled by the flexible nitrogen phosphorus backbone.^67^ However, Polymer 2 is modified by the inclusion of crown ether groups, which have been shown to enhance ionic conductivity in other SPEs.^68,69^ Exploration polymers like Polymer 1 and 2, or others predicted to have high ionic conductivity, may yield promising new SPEs.

### Screening Anions to Investigate Role in Ionic Conductivity

**Figure 7:**
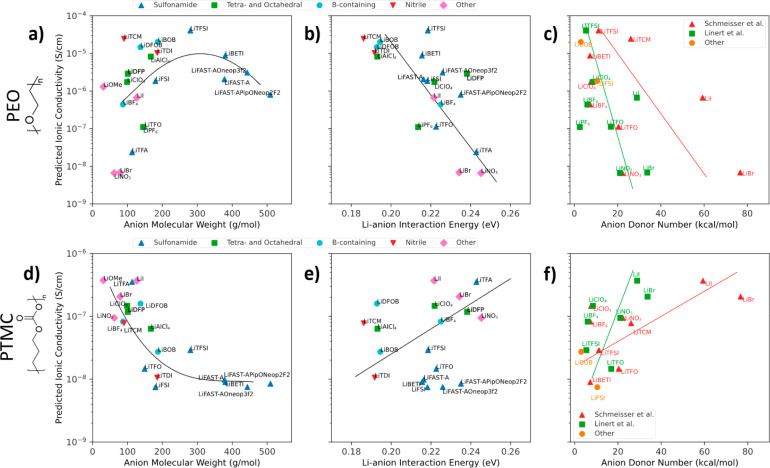
Predicted ionic conductivity for PEO (a–c) or PTMC (e, f) with various lithium salts at 1.5 mol of salt per kg of polymer and 25 °C. (a, d) Predicted ionic conductivity vs anion molecular weight. (b, e) Predicted ionic conductivity vs lithium–anion interaction energy calculated with DFT. (c, f) Predicted ionic conductivity vs anion donor number. Experimental values for anion donor number were extracted from Schmeisser et al.,^70^ Linert et al.,^71,72^ or other sources^73,74^ with points being colored accordingly. The different donor number values and slopes with respect to predicted ionic conductivity reflect different methods of measuring anion donor number in different sources. See Figure S24 for chemical structures of the anions shown above.

ChemArr was also used to explore the role of the anion in ionic conductivity. Figure 7 shows the predicted ionic conductivity of PEO or poly(trimethylene carbonate) (PTMC) with various lithium salts. As the model has been trained on many examples of PEO and PTMC SPEs, we expect the model predictions to be highly accurate for these polymers (see Figure S6). This allows us to make predictions while fixing certain parameters so we can examine trends that otherwise might be obscured. In this case, the polymer molecular weight, salt concentration, and temperature are kept constant while varying only the anion chemistry. The salt concentration was fixed at 1.5 mol of salt per kg of polymer because this is well within the range of concentrations reported for these salts in the literature (Table S7) and near the peak ionic conductivity reported for many systems in our database. We expect that the salts for which we do not have data will still be soluble in these two polymers at the selected concentration.

Figures 7a shows predicted ionic conductivity vs anion molecular weight for PEO. A volcano trend emerges, with anions of near 300 g/mol showing the highest predicted ionic conductivities. Several reports have demonstrated that large anions with distributed charge dissociate more freely from the lithium cation, thereby enhancing ionic conductivity,^75−77^ but the enhancement appears to disappear as the anion grows larger than 300 g/mol, likely due in part to decreased contributions to ionic conductivity from large, less diffusive anions. Large anions may also have adverse effects on cation solvation structure^78^ or polymer dynamics, an effect which has not been well studied for SPEs. Figure 7b shows ionic conductivity predictions plotted against the lithium–anion interaction energy calculated with DFT. Here, a relatively strong negative trend emerges. Figure 7c shows ionic conductivity predictions vs anion donor number, an experimental measure representative of interaction strength between the anion and a positive charge.^70^ Although different sources report different donor numbers, the negative correlation between predicted ionic conductivity and anion donor number is consistent across multiple sources, confirming the trend seen in our DFT calculations. This trend has been reported previously.^71,79,80^ Taken together, panels a–c of Figure 7 suggest that an ideal anion to enhance ionic conductivity in PEO would have a low interaction strength and a molecular weight between 200 and 300 g/mol. The fact that TFSI already meets these criteria suggests that TFSI may already be close to optimal for PEO SPEs. However, anions containing nitrile or boron groups, such as lithium tricyanomethanide (LiTCM) and lithium bis(oxalato)borate (LiBOB), show promising ionic conductivity and have not been studied as extensively as LiTFSI-like salts. Further study with or modification to these anions may result in anions yielding equal or higher ionic conductivity in PEO as for LiTFSI.

Panels d–f of Figure 7 show the results of predicting ionic conductivity for PTMC with the same anions as in Figure 7a–c. Interestingly, the trends seen in the case of PEO are reversed when the same anions are paired with PTMC, although the trend in Figure 7e is not as strong as that of Figure 7b. For PTMC, the ChemArr model predicts higher ionic conductivities for smaller anion molecular weights and higher lithium–anion interaction strengths. The different trends in ionic conductivity vs anion molecular weight and interaction strength in the PTMC-based electrolyte likely result from differing coordination strength or solvation structure^81,82^ of PTMC compared with PEO. It may be that smaller, more strongly interacting anions interact favorably with the carbonyl oxygens in PTMC, which individually bind more tightly to the lithium ion than the ether oxygens in PEO, or that the stronger coordinating ability of carbonyl carbons is synergistic with the formation of weak contact ion pairs.^81^ As the data shown in Figure 7 were generated with machine learning predictions, there will be errors associated with the ionic conductivity predictions. Further experimental and simulation work would be valuable to confirm the trends shown and to identify the underlying mechanistic causes of the different trends in PEO and PTMC.

## Conclusions

In this work, a chemistry-informed neural network was developed to accurately predict ionic conductivity in solid polymer electrolytes. Our model, ChemArr, incorporates the Arrhenius equation to give significantly improved prediction accuracy over models without embedded chemistry. ChemArr was trained on a data set of polymer electrolyte ionic conductivity gathered from over 200 experimental publications and gives predictions at or near experimental accuracy for most of the SPEs in our data set. We screened over 20,000 potential SPEs and identified polymer chemistries of interest for further characterization, allowing us to guide experimental efforts to promising systems, which could result in more effective use of experimental resources. We also investigated the effect of varying the anion in PEO and PTMC electrolytes. We found for both polymers that the anion mass and interaction strength were correlated with the predicted ionic conductivity of an SPE. Interestingly, we found that while anions with moderately high mass and low interaction strength were favored in PEO, in the case of PTMC, anions with lower mass and higher interaction strength were favorable to enhanced ionic conductivity, a finding which warrants further study.

Overall, this work demonstrates the value of chemistry-informed ML to improve prediction accuracy and generalizability for materials property predictions in SPEs. We anticipate that ChemArr can also be extended to model any process that follows Arrhenius-type temperature dependencies. This approach of incorporating known physical equations or parameters into machine learning models promises to generalize to prediction tasks in a variety of domains, especially in fields where limited data sets can be supplemented with governing equations or constraints derived from previous scientific knowledge.

## Data Availability

The computational models and polymer electrolyte data reported in this work are available under the MIT license at https://github.com/learningmatter-mit/Chem-prop-pred.

## Supporting Information Available

Details on experimental data extraction, performance of variations of machine learning models, error estimation, and validation of machine learning predictions with experimental data (PDF)
