# Supplementary material for: Chemistry-Informed Machine Learning for Polymer Electrolyte Discovery
Source: ACS Cent Sci. 2023 Jan 23;9(2):206–16. doi: 10.1021/acscentsci.2c01123 (PMC9951296; doi:10.1021/acscentsci.2c01123)
Supplement: Supplementary file 1 — oc2c01123_si_001.pdf [file oc2c01123_si_001.pdf]

Supporting Information for

Chemistry-Informed Machine Learning for Polymer Electrolyte Discovery

Gabriel Bradford[1], Jeffrey Lopez[2], Jurgis Ruza[4], Michael A. Stolberg[3,4], Richard Osterude[4], Jeremiah A. Johnson[3], Rafael Gomez-Bombarelli[4]*, and Yang Shao-Horn[1,4]*

Author Affiliations:
[1] *Department of Mechanical Engineering, Massachusetts Institute of Technology, 77 Massachusetts Ave, Cambridge, MA 02139, USA.*

[2] *Research Laboratory of Electronics, Massachusetts Institute of Technology, 77 Massachusetts Avenue, Cambridge, Massachusetts 02139, USA*

[3] *Department of Chemistry, Massachusetts Institute of Technology, 77 Massachusetts Ave, Cambridge, MA 02139, USA.*

[4] *Department of Materials Science and Engineering, Massachusetts Institute of Technology, 77 Massachusetts Ave, Cambridge, MA 02139, USA*

*corresponding author

email: R.G.B. – rafagb@mit.edu, Y.S.H. – shaohorn@mit.edu

Contents

Experimental dataset extraction and formatting S3
Data quality checks S3
Format of SPEs in dataset S3
Distributions of materials in ionic conductivity dataset S3
Machine learning performance with input and architecture variations S5
Model performance with different salt representations S5
Model performance with lithium-polymer interaction energy input S6
VTF model performance S6
Effect of balancing PEO S7
Error estimation S9
Model accuracy for prediction on PEO based electrolytes S10
Model improvement with increasing training data S11
Spearman R calculation S12
Comparison of machine learning model with previous work S13
Effect of polymer chemistry on ionic conductivity S14
Validation of ML predictions with known experimental trends S16
Verification of improved temperature dependence of ChemArr model S19
Experimental electrolyte preparation and conductivity measurement of PEO-LiTDI system S20
Synthesis of novel precursors, monomers, and polymers S21
Chemical structures of anions screened with PEO and PTMC S28

Experimental dataset extraction and formatting

Data quality checks

Table S1 shows the specific checks that were made to publications before extracting polymer electrolyte ionic conductivity data. Each paper was checked by a graduate student or post-doc in the Shao-Horn lab after which data was manually collected for qualifying papers. Data was aggregated into a dataset used for training our machine learning models.

Table S1

	**Data Extraction Checklist**
1.	Water free sample preparation and conductivity measurement
2.	Drying conditions specified or residual solvent reported
3.	Sample preparation and measurement in glovebox
4.	Polymer molecular weight reported
5.	Experimental data contains reasonable trends and errors
6.	No general red flags on sample preparation or conductivity measurement

Format of SPEs in dataset

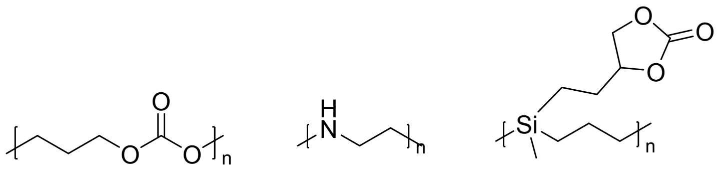

[Au]CCCOC(=O)O[Cu] [Au]NCC[Cu] [Au][Si](C)(CCC1OC(=O)OC1)CCC[Cu]

Figure S1: Examples of SMILES representations of polymers

In our dataset, each polymer was represented with the smiles string of the monomer unit, using "[Au]" and "[Cu]" caps to signal the end of the monomer unit (see Figure S1 for examples). Au and Cu were chosen since they did not appear in any of the original polymers. In the case of co-polymers, each co-monomer unit was stored, as well as the molar ratio between them. Similarly, for polymer blends or polymer-liquid mixtures each component of the mixture was represented with a smiles string and molar and weight percentages were stored to specify the composition.

Distributions of materials in ionic conductivity dataset

Figure S2 shows the distributions of all solvents, the polymers, and additives in the experimental dataset. The experimental data is skewed towards PEO (SMILES: "[Cu]OCC[Au]") and PTMC (SMILES: "[Cu]OC(=O)OCCC[Au]"). There are roughly 4,500 datapoints for PEO-based

electrolytes and around 1,000 datapoints for PTMC-based electrolytes. Aside from these two polymers, the rest of the data is relatively evenly distributed among the remaining 200+ polymers in the dataset, many of which have ~100 datapoints. Although we did not use liquid electrolyte data in this work, we collected over 3,500 datapoints of liquid electrolyte ionic conductivity where conveniently available from the Hatakeyama-Sato et al. dataset[1] or from publications containing both polymer and liquid electrolyte data. For our learning task, all liquid solvent data was ignored, removing a few thousand datapoints from liquid electrolytes.

Given the skew of the data towards PEO, we attempted to balance the training data to achieve better performance when predicting on non-PEO polymers. To balance the training data, we augmented all non-PEO by duplicating all data until it reached a threshold number of datapoints, which was chosen relative to the number of PEO datapoints. In the first case we duplicated data until each polymer had 1/5 as much data as the PEO data. In the second case we duplicated data until each polymer had ½ as much data as PEO. To avoid too much repetition of the same data, we also impose a limit on the number of repeats at 20, so no datapoint could be repeated more than 20 times. For some polymers, this meant the total amount of data was less than 1/5 or ½ of the PEO data. Table S5 shows the results of training the model on these different training sets. The error metrics reported are for a test set composed of all non-PEO polymers. Somewhat surprisingly, neither data augmentation scheme gave an improvement in performance. This is likely caused by the model overly learning some random noise in the data since it is trained on repeats of the same data.

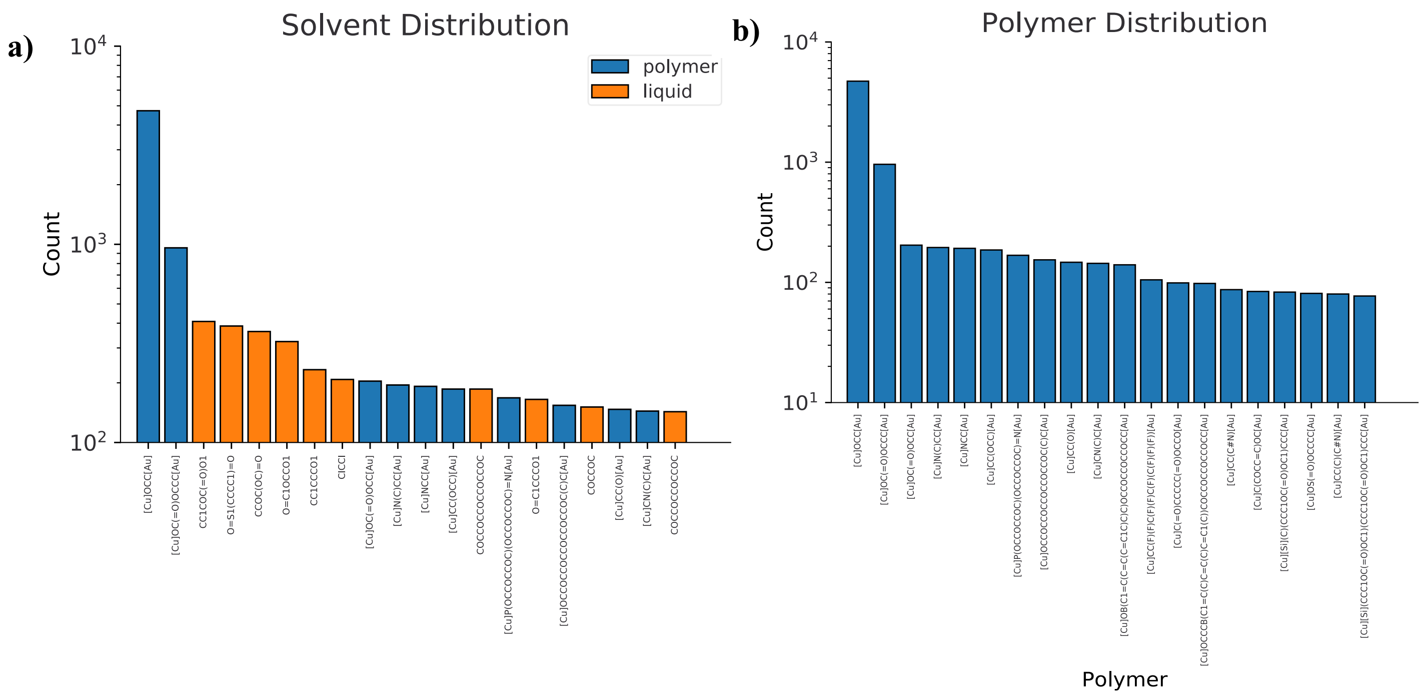

Figure S2: Distributions of a) solvents and b) polymers in the experimental dataset.

Machine learning performance with input and architecture variations

Table S2: Performance metrics for each of the models tested excluding predictions for all datapoints with experimental ionic conductivity <10^{-4} S/cm.

	Mean Absolute Error (log(S/cm))	Spearman R
**XGBoost (high cond)**	0.877	0.24
**Chemprop (high cond)**	0.734	0.38
**Chemarr (high cond)**	0.63	0.44

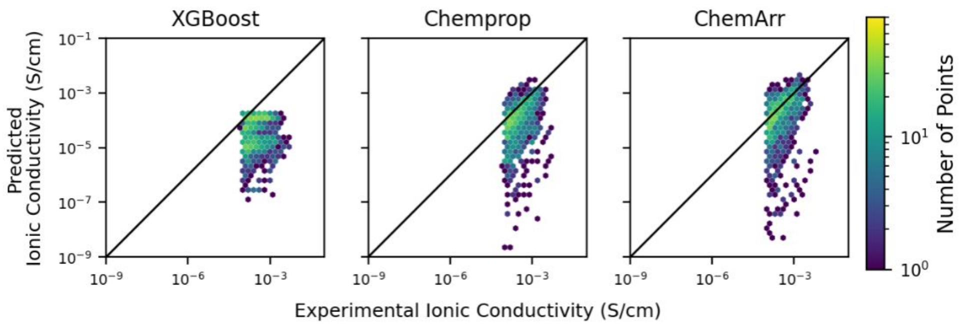

Figure S3: Predicted vs experimental ionic conductivity on cross-validation (see Methods) for XGBoost, Chemprop, and ChemArr excluding predictions for all datapoints with experimental ionic conductivity <10^{-4} S/cm.

Model performance with different salt representations

Model performance was evaluated using a few different methods to represent salt structures in SPEs. In each case, the polymer was featurized using the MPNN from Yang et al.[2] One salt representation was to calculate the Morgan fingerprint vector of the salt, which was then concatenated to the learned feature vector for the polymer. Another method was to featurize the salt with a MPNN separately from the polymer, so each species was individually passed through a different MPNN. In this case, each MPNN had different weights learned for either the polymer or salt. The final representation, which we used in all testing of the model, was to input the polymer and salt together as a disconnected graph into one MPNN, which learned a representation of both species at once. Performance for each of these methods is shown in table S2.

Table S3

Salt Representation	Mean Absolute Error	Spearman R
Salt Morgan FP	1.09 +- 0.073	0.53 +- 0.066
Salt Separate MPNN	1.04 +- 0.029	0.58 +- 0.019
Poly-Salt MPNN	**1.00 +- 0.030**	**0.59 +- 0.022**

Model performance with lithium-polymer interaction energy input

We trained our model using the interaction energy between the polymer and lithium as an additional feature. Interaction energies were calculated using density functional theory (DFT). To calculate the interaction energy, **x** conformations of a 50 atom oligomer chain with a Li+ ion were generated. For each, the total energy of the complex was calculated. The lowest energy complex was then selected, and the energy of the Li+ without the polymer and the polymer without the Li+ were calculated. The interaction energy was then calculated according to the equation $E_{interaction} = (E_{polymer} + E_{Li+}) - E_{polymer-Li+}$, where $E_{polymer}$, E_{Li+}, and $E_{polymer-Li+}$ are the values calculated in DFT. Energies were calculated with the wB97XD functional. Table S4 shows the model performance with and without the interaction energy. The model trained on the interaction energies gives slightly better performance, although both models fall within the same range of error. Given the small improvement and computational cost of calculating the interaction energy, we decided not to include interaction energy as a feature in the final model.

Table S4

**Model**	**Mean Absolute Error**	**Spearman R**
Without DFT interaction energy	1.00 +- 0.030	0.59 +- 0.022
With DFT interaction energy	**0.98 +- 0.041**	**0.63 +- 0.023**

VTF model performance

The Vogel-Fulcher-Tammann (VFT) equation, like the Arrhenius equation, can be used to describe ionic conductivity in polymer electrolyte. The log form of the equation is $\ln(\sigma) = \ln(A) - \frac{E_a}{R(T-T_0)}$ where σ is ionic conductivity, A is the prefactor, E_a is activation energy, R is the ideal gas constant, T is the temperature, and T_0 is the fitting temperature. The addition of T_0 adds flexibility beyond the Arrhenius equation fit, allowing the VFT equation to describe ionic conductivity behavior that doesn't strictly follow Arrhenius behavior. If $T_0 = 0K$, the VFT equation is the same as the Arrhenius equation. We attempted to develop a model based on the VFT equation rather than the Arrhenius equation. The VFT model was built the same as the model described in the main text, with the exception that the final layer estimated the three parameters of the VFT equation, $\ln(A)$, E_a, and T_0. These parameters were then used to calculate the conductivity according to the VFT equation. The VFT model was trained on the same data as with the Arrhenius model, and hyperparameters were optimized with hyperopt. However, the VFT model failed to learn VFT behavior in the polymer electrolytes, giving T_0 outputs near 0K in all cases. Figure S4a shows a histogram of the outputs of a trained VFT model for T_0. The outputs are all slightly negative near 0K. Figure S4b shows the distribution of the fitted T_0 terms for the same data, which gives a bimodal distribution with peaks at 0K and 200K. The VFT model we trained was not able to learn T_0 values higher than zero. This is likely due to the relatively small increase in performance given by learning T_0 correctly. The Arrhenius

fit gives a good approximation for most electrolytes in our data, with only some examples demonstrating strongly non-Arrhenius behavior over a sufficient temperature range for the VFT fit to deviate significantly from the Arrhenius fit. The model likely defaults to $T_0 \approx 0K$ because of this.

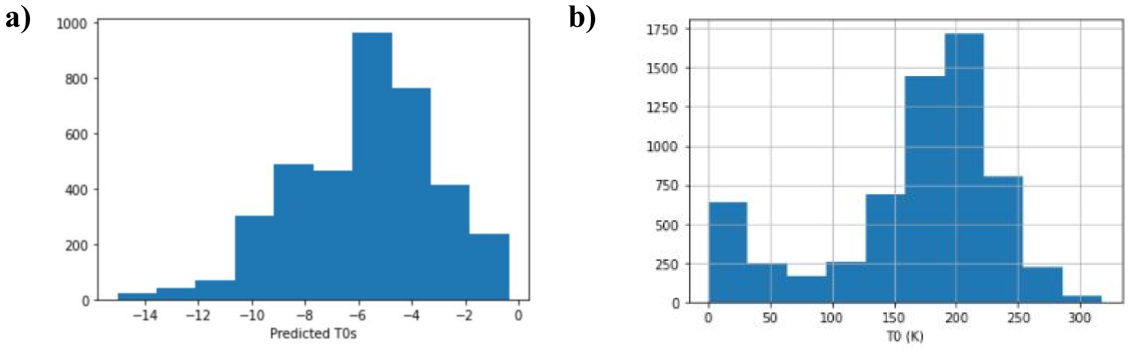

Figure S4: a) Histogram of predicted T_0values from the VFT model. b) Histogram of T_0 values fitted from the experimental training data.

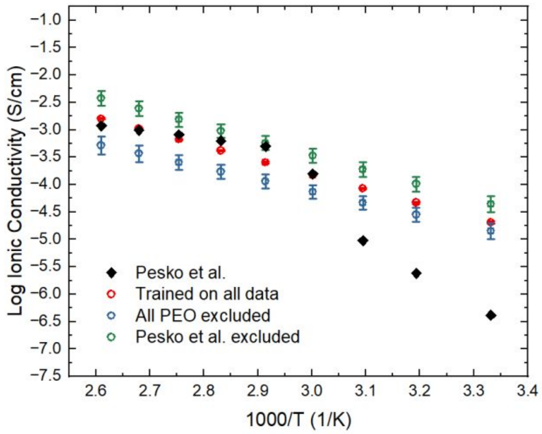

Figure S5: ChemArr predictions compared to literature data for 0.45 mol LiTFSI per kg PEO from Pesko et al.[3] Predictions were made using models trained on three different datasets, one using all available data, one excluding all PEO data, and one excluding on the data from the paper containing the data being compared against. All three predictions fail to capture the dual slope behavior of the PEO electrolyte that results from different activation energies above and below the melting point.

Effect of balancing PEO

As is seen in Figure S2b, polymer electrolytes based on poly(ethylene oxide) (PEO) are most common in the experimental dataset. This results from decades of experimental focus on PEO as a polymer electrolyte matrix based on its good ability to solvate lithium salts and flexible backbone units promoting ion diffusion.[4] Given the depth of experimental study for PEO, data

for PEO based electrolytes is more common than for other polymers, with PEO accounting for nearly 30% of our training data. We attempted a few data balancing methods to see if model performance would improve with more balanced training data. When performing these tests, we followed the same cross-validation procedure outlined in Methods of the paper, with PEO kept out of all test sets, therefore measuring the model's predictive ability on non-PEO polymers.

In our data balancing, we augmented the non-PEO data by replicating datapoints in the dataset until the number of datapoints for the polymer were equal to some fraction of the number of PEO data points. The fractions we measured were ½ PEO and 1/5 PEO. For example, if PEO had 3,000 data points and Polymer X had 100 datapoints and we were replicating to ½ PEO, each datapoint for Polymer X would be replicated 15 times to give 1,500 datapoints for Polymer X. This involves some obvious shortcomings from repeating the same data multiple times but allowed us to balance the training data without removing PEO data that could contain valuable information.

Table S5 summarizes the results of data balancing with ½ and 1/5 PEO levels. Leaving the data as is, with no balancing, gives the best performance and is the method we followed for our ultimate predictions. While this is a bit surprising, it may be because the PEO contains much greater diversity in terms of salts and concentrations than other polymers, so the PEO data allows the model to learn trends that are hard to learn from other chemistries.

Table S5

**Training data balancing**	Mean Absolute Error	Spearman R
Augmented to 1/5 of PEO	1.04 +- 0.063	0.57 +- 0.056
Augmented to ½ of PEO	1.03 +- 0.030	0.55 +- 0.030
**No Balancing**	**1.00 +- 0.030**	**0.59 +- 0.022**

Error estimation

The error estimation used for the model leverages the test set error to estimate error for future predictions. For each datapoint in the test set, a weighted average Tanimoto distance was calculated between the datapoint and the training data, using the equation

$$d = 1 - \frac{1}{N} \Sigma \, \mathrm{e}^{-\frac{\mathrm{a(TD)}}{1-\mathrm{TD}}}$$

from Liu et al.[5] where d is the average distance, a is a weighting factor that changes how distance is weighted between closer and further species, and TD is the Tanimoto distance between two molecules. Tanimoto distances were calculated using Morgan fingerprints of polymers and salts. We used a=3 to calculate the distance, according to results from Liu et al.

With each test datapoint having both a prediction error and distance value, the expected error for a new predicted datapoint can be estimated from the test errors and distances. This was done by first calculating the distance between the new datapoint and the training data. Then, the errors for every test point with a chemical distance within 0.05 of the new point's chemical distance are collected. (A distance of 0.05 was chosen to ensure at least 100 datapoints were used to estimate the error.) Then, each of these errors is used to calculate a "standard deviation" at that specific distance value, according to the formula $\sigma = \sqrt{\frac{\Sigma \epsilon^2}{N}}$, where ϵ is the error for each datapoint and N is the number of datapoints. The standard deviation can then be used to estimate the width of a confidence interval around each prediction.

Model accuracy for prediction on PEO based electrolytes

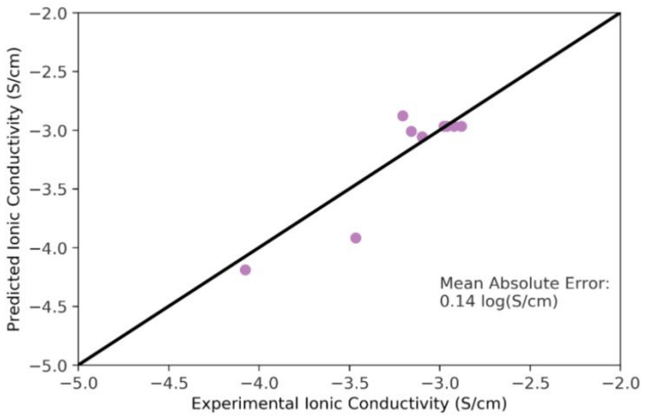

Figure S6: Predicted vs experimental ionic conductivity for PEO with various lithium salts at 80 °C and 1.5 molality.

Figure S6 shows the correlations between predicted and experimental values of PEO electrolytes at 80°C and 1.5 molality. Data at 80°C is shown since it is the temperature where there are the most datapoints for PEO at exactly 1.5 molality. Even so, there are relatively few datapoints in our experimental set at exactly 80°C and 1.5 molality, limiting the number of points in the figure. The data shown are for several lithium salts, namely LiTFSI, LiBETI, $LiPF_6$, LiTFO, LiTDI, and LiTCM. The mean absolute error for these predictions is 0.14 log(S/cm), which is well within the range of experimental error. This gives confidence that our model predictions for PEO systems will be near experimental accuracy, supporting our ability to examine trends in ionic conductivity for PEO-based systems.

Model improvement with increasing training data

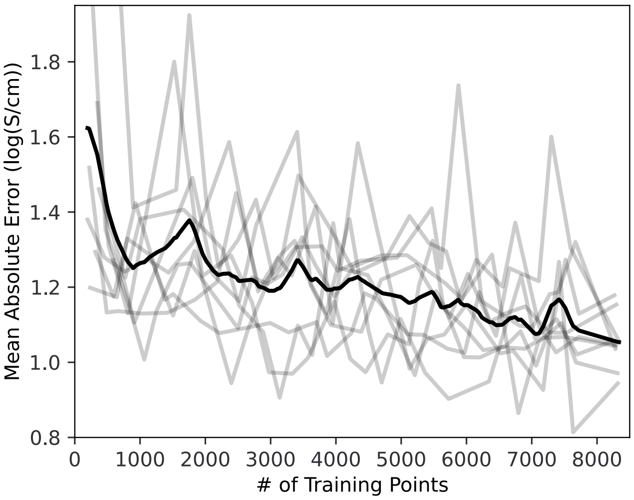

Figure S7: Mean absolute error of predictions vs number of datapoints in the training set. The error is calculated for predictions on a test set of polymer structures removed from the training data. The results of 10 independent runs with different randomly selected test sets are shown in light grey while the average is shown in black. For each run, the model was successively trained in increasingly larger training sets, then its prediction accuracy was measured on the test set. Data was added in batches to replicate experimental discovery, where a batch consisted of all data associated with 12 SPE formulations in our dataset.

While our ML model's predictions are more accurate than the other models we tested, Figure S7 suggests that the model's accuracy will improve with more training data. Figure S7 shows that the prediction error decreases as the size of the training set increases. Notably, the rate of decrease in prediction error doesn't appear to plateau as we reach our maximum amount of training data, indicating that model could improve further given more experimental data. Efforts to increase the availability of ionic conductivity measurements would improve our model's ability to accurately predict ionic conductivity for a range of SPEs. Automated extraction of values from existing literature or a system of data sharing among researchers working on ionic conductivity experiments would be valuable to advance our ability to both predict ionic conductivity in novel SPEs and understand trends from existing data. Obtaining data on previously untested or rarely tested SPE formulations will lead to improvements in prediction accuracy across the polymer space and yield insights into drivers of ionic conductivity in SPEs.

Spearman R calculation

Since ChemArr explicitly encodes ionic conductivity temperature dependence via the Arrhenius equation, direct comparison of Spearman R for the different models' predictions would unfairly favor ChemArr over models which do not encode temperature dependence. The Spearman R values for ChemArr would be inflated since by design our model gives lower ionic conductivity values at lower temperatures, therefore improving its ranking performance for datapoints at different temperatures in the same electrolyte.

To avoid unfair comparison between ChemArr and the other models tested during benchmarking, we calculated the Spearman R value separately for ionic conductivity data at ten temperatures ranging from 10 to 100°C. We then averaged the Spearman R values from each temperature to get a total Spearman R value for each model. This ensured a fair comparison of Spearman R values between the models.

Comparison of machine learning model with previous work

During the development of this work, Hatakeyama-Sato et al.[1] released a dataset of polymer electrolyte ionic conductivity as well as machine learning results for learning on that dataset. We compared our model performance Hatakeyama-Sato et al.'s model on the training and test sets used in Hatakeyama-Sato et al.'s work.

Figure S8 shows parity plots for Hatakeyama-Sato et al.'s model and our model, where we imitated the formatting of the original plot. Table S6 shows the mean absolute error and R^2 on the test set data for both models. Hatakeyama-Sato et al. did not report a specific value of mean absolute error for their model, but state that it is below 0.8. Our model performs better for both metrics than the previous work. Both models struggle with a group of electrolytes with experimental conductivities around -2 log(S/cm), but our model gives predictions about an order of magnitude closer to the experimental values than the model from Hatakeyama-Sato et al. The better performance of our model on this data than our dataset primarily reflects that the test set from Hatakeyama-Sato et al. contained polymers in the training set as well, whereas our model was benchmarked on test sets composed of polymers excluded from the training set.

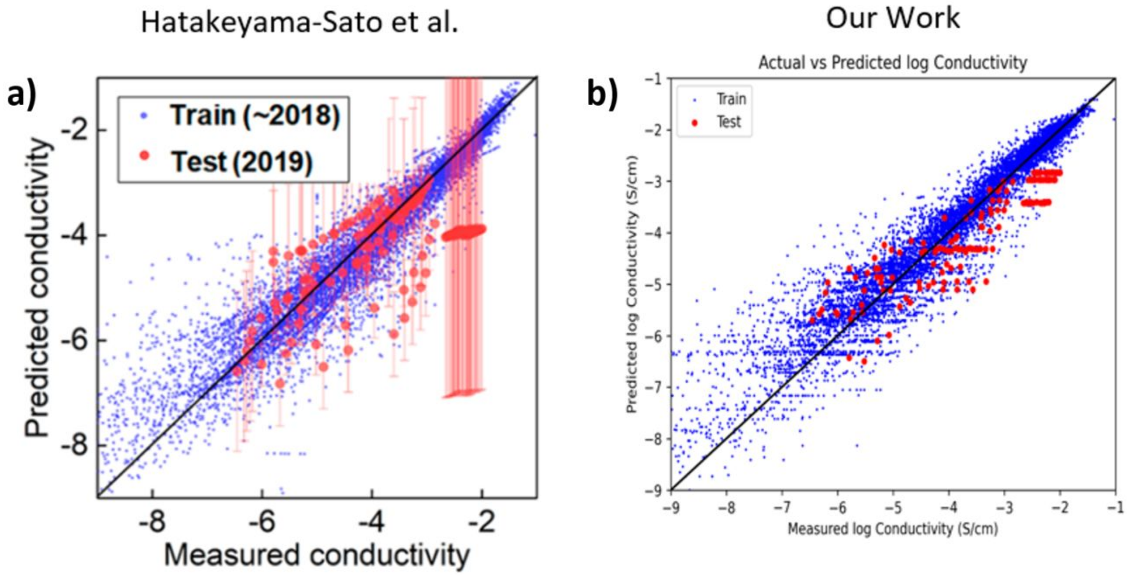

Figure S8: Predicted vs measured (experimental) ionic conductivity from a) Hatakeyama-Sato et al.[1] and b) our model on data from Hatakeyama-Sato et al.

Table S6

	Mean Absolute Error (log (S/cm))	R^2
Hatakeyama-Sato et al.	< 0.8	0.16
**Our Work**	**0.59**	**0.69**

Effect of polymer chemistry on ionic conductivity

Using data from both our experimental dataset and predictions from our model, we explored the effects on chemical structure on ionic conductivity. Figure S9 shows distributions of the experimental data divided by a) percentage of oxygen, nitrogen, or sulfur (N,O,S) in the polymer or b) percentage of atoms in the polymer backbone, as opposed to atoms in side chains. For both measurements, hydrogen atoms are ignored. Figure S9a shows little trend in ionic conductivity with varying percentage of N,O,S. Figure S9b perhaps shows lower ionic conductivity values at intermediate backbone percentages, with higher values at very low and very high percentages. This may be a result of specific experimental focus on both linear PEO-like polymers and polymers with long PEO-like side chains that coordinate and transport Li+ ions, giving rise to higher conductivities for both polymers with large sidechains and linear polymers.

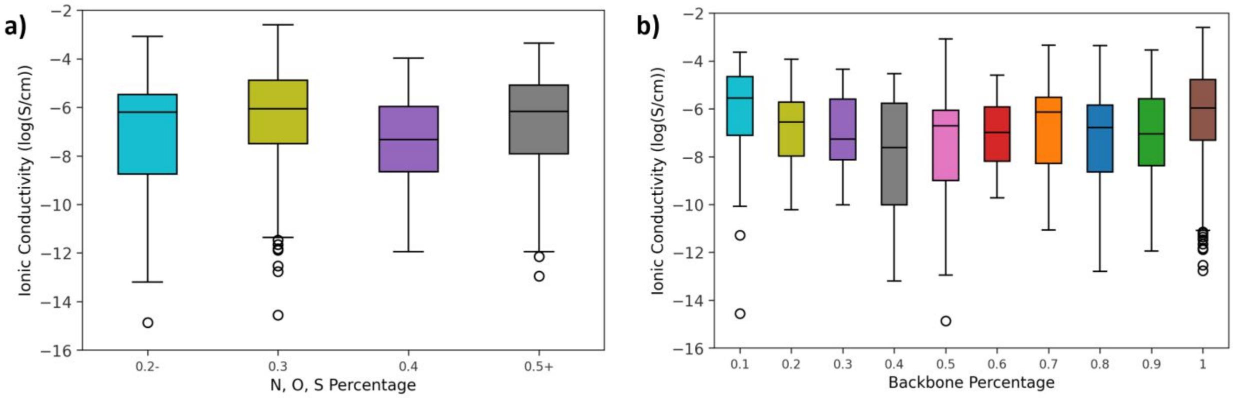

Figure S9: a) Ionic conductivity distributions for discrete percentage compositions of nitrogen, oxygen, and sulfur. b) Ionic conductivity distributions for backbone percentage, or the percentage of the total atoms found in the polymer backbone, excluding hydrogen. Data shown in both plots comes from the experimental data gathered, where ionic conductivities were interpolated to 80°C using an Arrhenius fit of existing data.

Figure S10 shows similar plots as in Figure S9, but the data used to generate these plots comes from our machine learning model predictions on 756 distinct polymers combined with 8 lithium salts to give nearly 20,000 polymer electrolyte formulations. Like Figure S9a, Figure S10a shows no trend in ionic conductivity as a function of the N,O,S percentage. Figure S10b, however, shows a general increase in ionic conductivity as the backbone percentage increases above 0.8. The reason for the difference in trends between Figure S9b and S10b likely results from the different type of side chains prevalent in each dataset. The experimental data shown in Figure S9b contains nearly exclusively side chains with coordinating ether or carbonyl groups. The data shown in Figure S10b contains relatively few side chains with such coordinating groups, with many side chains composed purely of carbon or aromatic rings. These non-coordinating side chains inhibit ion transport and reduce the overall ionic conductivity in the polymer.

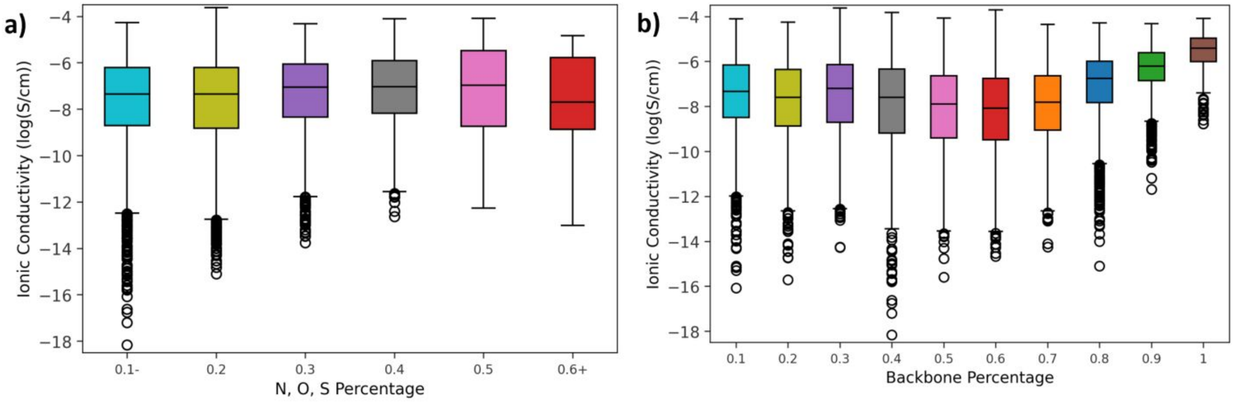

Figure S10: a) Ionic conductivity distributions for discrete percentage compositions of nitrogen, oxygen, and sulfur. b) Ionic conductivity distributions for backbone percentage, or the percentage of the total atoms found in the polymer backbone, excluding hydrogen. Data shown in both plots comes our machine learning model predictions on ~20,000 hypothetical polymer electrolytes.

Validation of ML predictions with known experimental trends

In the polymer electrolyte literature, there a several well-known trends in ionic conductivity as a function of different parameters. We examined three published trends to ensure the ChemArr model predictions follow the trends we expect from experiments. Each plot in Figure S11 shows data for three polymers: PEO, PTMC, and PSBMC, the structures of which are shown in Figure S12. Figure S11a shows the predicted ionic conductivity vs salt concentration. One of the complexities of predicting ionic conductivity for a wide range of polymer electrolytes is that different polymers give rise to different trends in ionic conductivity vs salt concentration. However, the model captures the trends seen experimentally,[6–8] an indication of good learning by the model. Figure S11b shows the predicted ionic conductivity vs polymer molecular weight for the same systems. The trend shows a sharp decrease as polymer molecular weight initially increases from a low value of 100 g/mol, then levels out as the molecular weight surpasses around 50,000 g/mol. This trend matches what has been reported experimentally.[9,10]

Figure S11c shows the lithium diffusivity vs salt concentration in molarity. Since diffusivity is often not reported in the experimental data gathered to train the model, we are unable to predict diffusivity directly. However, it can be calculated with the equation

$$D^{+} = \frac{\sigma t_{+} RT}{c(ZF)^2}$$

where D^{+} is the lithium diffusivity, σ is the total ionic conductivity, t_{+} is the lithium-ion transference number, R is the ideal gas constant, T is the temperature, c is the salt concentration in units of moles per cm^3**,** Z is the lithium-ion valence, and F is Faraday's constant. $R, T, Z,$ and F are all known constants. To calculate D^{+}, then, we estimate $t_{+} = 0.3$ based on reported values.[11,12] To calculate c, we use the equation

$$c = \frac{\rho_1}{1 + \frac{1000\rho_1}{A(MM_1)\rho_2}},$$

where ρ_1 is the salt density, ρ_2 is the polymer density, A is the molality, and MM_1 is the salt molar mass. Although this analysis does include an assumption about the transference number, and an approximate calculation for concentration in terms of molarity, the trends we see in figure S11c for PEO are similar to what has been reported experimentally.[13]

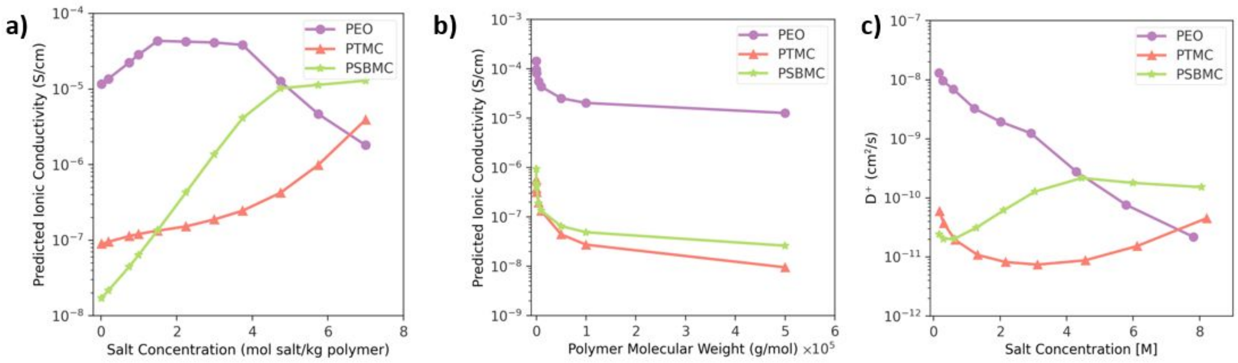

Figure S11: Predicted ionic conductivity vs a) salt concentration and b) polymer molecular weight for PEO, PTMC, and PSBMC. c) Lithium diffusion coefficient vs salt concentration for PEO, PTMC, and PSBMC. In each case, predictions were made with LiTFSI as the salt and a temperature of 25˚C

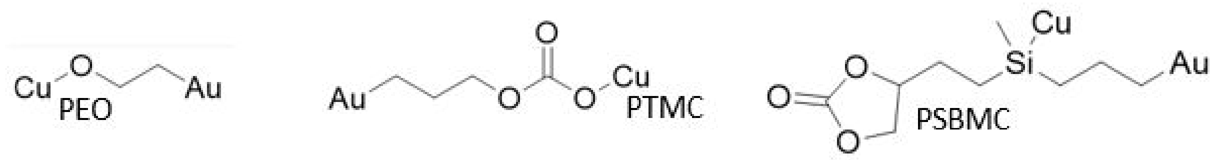

Figure S12: Polymer structures for PEO, PTMC, and PSBMC. "Cu" and "Au" are placed the ends of the monomer units and do not represent real elements in the monomer.

An additional trend that has been reported previously is the entropy enthalpy compensation, a phenomenon seen in a variety of systems. Figure S13 shows that the ChemArr model learns the entropy enthalpy compensation from the training data, leading to a roughly linear relationship between the predicted activation energy and log of the prefactor. This relationship was not encoded into the model in any way, rather it was learned from the training data. Figure S14 shows a similar plot for the experimental data gathered, which shows a similar linear relationship between the activation energy and log of the prefactor.

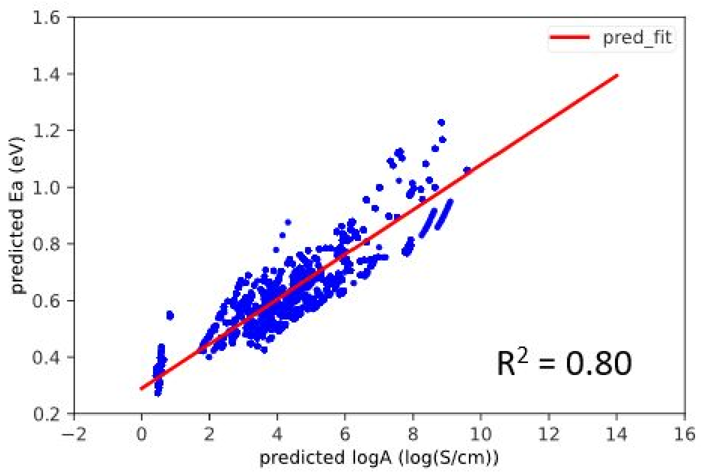

Figure S13: Predicted activation energy vs predicted log of the prefactor of the Arrhenius equation. The red line is a linear fit of the blue data points. All blue data points on the plot are from test set predictions of the model.

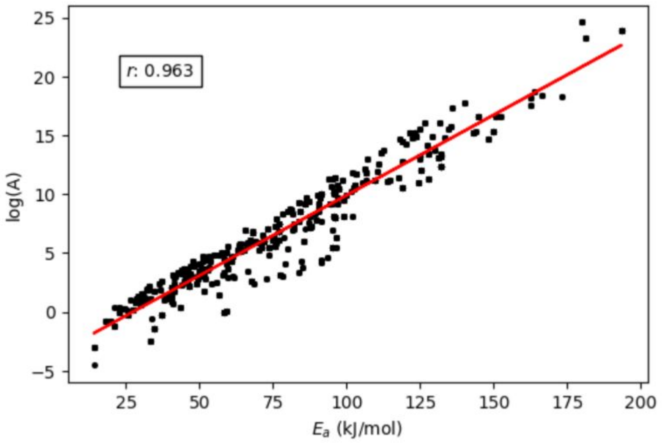

Figure S14: Log of the prefactor vs activation energy for all polymer electrolytes in the experimental dataset. The red line is a linear fit through the data.

Verification of improved temperature dependence of ChemArr model

To verify the effect of encoding the Arrhenius equation in the ChemArr model, we compared predictions on a set of PEO with LiTDI data before and after adding the Arrhenius constraint. Figure S15 shows a heatmap of experimentally obtained ionic conductivity values at various temperatures and concentrations. Data was obtained as described in the following section.

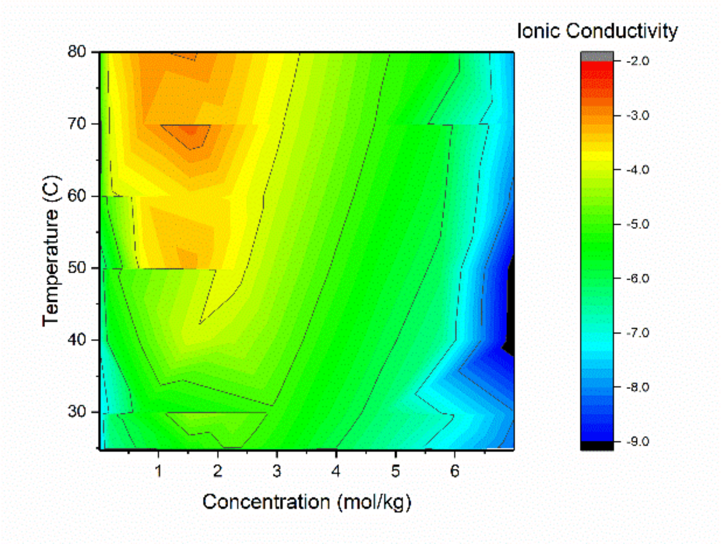

Figure S15: Heat map of ionic conductivity of PEO-LiTDI system at various concentrations and temperatures.

Figure S16a shows predicted ionic conductivity values for the same PEO-LiTDI formulations using the Chemprop model before inclusion of the Arrhenius equation. The model is able to capture the general trends of the experimental data, but only captures about 2 orders of magnitude of difference, rather than about 6 orders of magnitude as shown in Figure S15. Figure S16b shows the predictions of ChemArr on the same data. While ChemArr still doesn't capture the full range of the experimental data, it does a much better job matching the trend of the experimental data, spanning nearly 4 orders of magnitude and better capturing the shape of the conductivity vs salt concentration trends. The predictions made in both Figures S16a and S16b were made using ML models that had never been trained on LiTDI data.

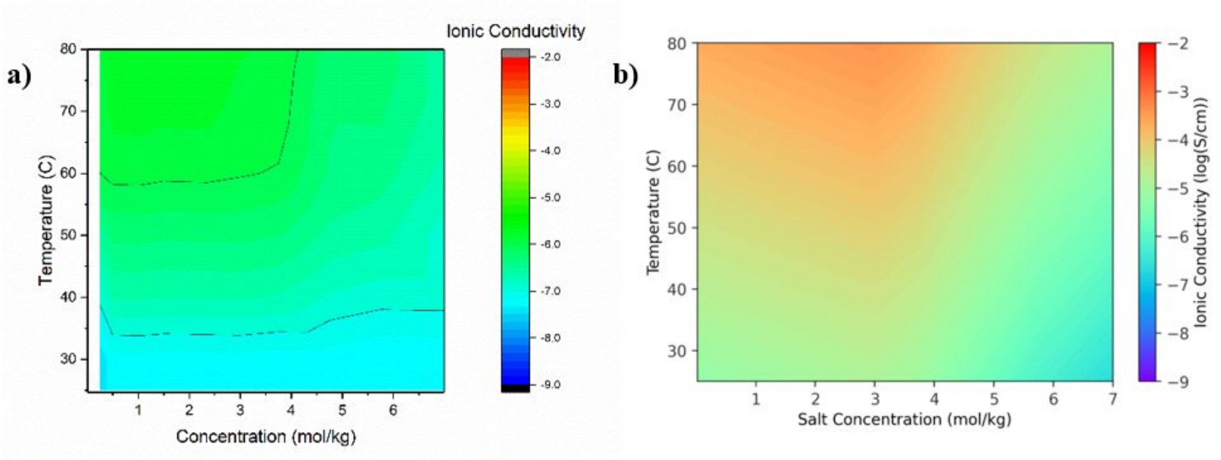

Figure S16: Heat maps of predicted ionic conductivity of PEO-LiTDI system at various concentrations and temperatures from a) Chemprop and b) ChemArr.

Experimental electrolyte preparation and conductivity measurement of PEO-LiTDI system

Poly (ethylene oxide) (M_V ~ 600,000 g/mol MilliporeSigma) and Lithium 4,5-dicyano-2-(trifluoromethyl)imidazolium (LiTDI) (provided by Arkema), were dried at 65°C under reduced pressure and then introduced to an argon filled glovebox without air exposure. Acetonitrile (MilliporeSigma) was dried over 4 angstrom molecular sieves. All subsequent operations were done in the glovebox and maintained at H_2O < 0.1 ppm and O_2 < 10 ppm. 0.75, 3.0 and 5.75 molality solutions of LiTDI in PEO powders were dispensed. The powders were then dissolved acetonitrile at a concentration of 70 milligrams of polymer per milliliter of solvent. The solutions were allowed to stir at 8 hours at 65°C to dissolve all the solids. The resultant solutions were cast onto stainless steel electrodes, and dried at 80°C under a gentle argon flow to remove acetonitrile resulting in polymer electrolyte films. A top stainless-steel electrode was added, and the sample was subjected to electrochemical impedance spectroscopy (EIS) from 80°C to 25°C. EIS was conducted on a Biologic VMP 300 over the frequency range of 1Hz to 7MHz, with a 50mV sinusoidal amplitude in a four-probe set up. EIS data were fit to an equivalent circuit model (See Figure S5). The ionic conductivity was calculated via the following equation,

$$\sigma = \frac{l}{R1 * A}$$

where l is the polymer film thickness, A is the contact area, and R1 is obtained from the fitted parameters of the model.

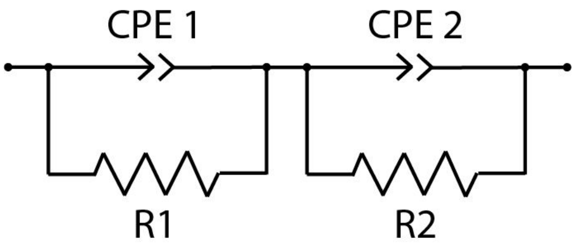

Figure S17: Equivalent circuit model used to fit EIS data. The equivalent circuit contains two in series sets of resistor and capacitor in parallel.

Synthesis of novel precursors, monomers, and polymers

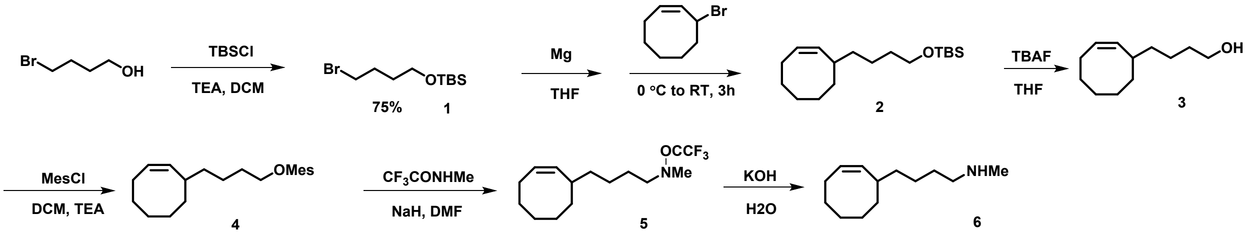

Figure S18: Reaction pathway for polymer precursor synthesis

1: To a stirred solution of 4-bromobutanol (11.5 g, 1 equiv) in dry CH_2Cl_2 (DCM, 60 mL), TBSCl (11.8g, 1.3 equiv), DMAP (0.05 equiv) and Et_3N (1.3 equiv) were added. The reaction solution was stirred at room temperature for 12 h, and then a saturated solution of NH_4Cl was added. The mixture was extracted with DCM and washed with brine and water. The organic layer was dried with Na_2SO_4 and concentrated. The residue was subjected to silica gel chromatography with petroleum ether/EtOAc (100:1) to give 1 as colorless liquid (11.0 g, 68%). ^{1}H NMR (400 MHz, Chloroform-*d*) δ 3.64 (t, *J* = 6.1 Hz, 2H), 3.45 (t, *J* = 6.8 Hz, 2H), 2.00 – 1.86 (m, 2H), 1.72 – 1.60 (m, 2H), 0.89 (s, 9H), 0.05 (s, 6H). ^{13}C NMR (101 MHz, Chloroform-*d*) δ 62.10, 33.49, 30.42, 29.03, 25.70, -2.95.

2: In a dry 250 mL flask, magnesium turnings (1.1 g, 1.2 equiv) that has been shaved to expose the fresh surface were mixed with a catalytic amount of iodine and 0.02 equiv **1** in 60 mL dried THF. The mixture was stirred for 15 min at 50 °C, followed by slow addition of **1** (10.2 g, 1.0 equiv) into the mixture over 3h by syringe pump. After the addition of **1**, the mixture was allowed to stir further for 2h at 50 °C and then cooled to room temperature.

To the solution of 3-bromo-1-cyclooctene (7.18 g, 1.05 equiv) and CuI (73 mg, 0.01 equiv) in dry THF (80 mL) under N_2 flow, the prepared Grignard reagent in THF solution was added dropwise via cannula needle at 0 °C over 0.5–1 h. The reaction mixture was allowed to warm to room temperature and stirred for 3 h. The reaction was carefully quenched by pouring into ice water and then neutralized with 2N HCl. The organic layer was separated, and the aqueous layer was extracted with diethyl ether three times. The combined organic layer was dried over anhydrous MgSO4. After concentration of the solution, the resulting crude product was acquired as a colorless liquid. The crude product was directly used for next step.

3: To a stirred solution of **2** (1 equiv) in 60 mL THF, 1.0 M TBAF (1.5 equiv) in THF was added. The reaction solution was stirred at room temperature for 24 h. Then THF was removed by vacuum and water was added to the mixture. The mixture was extracted with DCM and washed with brine and water. The organic layer was dried with Na_2SO_4 and concentrated. The residue was subjected to silica gel chromatography with DCM/hexane to give **3** as colorless liquid (5.9 g, 82% for two steps). ^{1}H NMR (400 MHz, Chloroform-*d*) δ 5.96 – 5.54 (m, 1H), 5.42 – 4.84 (m, 1H), 3.64 (q, *J* = 6.4 Hz, 2H), 2.43 (m, 1H), 2.20 (q, *J* = 11.3, 10.2 Hz, 1H), 2.02 (m, 1H), 1.76 – 0.99 (m, 14H). ^{13}C NMR (101 MHz, Chloroform-*d*) δ 135.47, 129.42, 62.84, 36.58, 36.50, 35.81, 32.85, 29.63, 26.92, 26.75, 25.83, 24.05.

4: To a 100 mL round-bottomed flask equipped with a magnetic stirring bar were added **3** (5.7 g, 1 equiv) and triethylamine (4.7 g, 1.5 equiv), followed by the addition of 50 mL freshly dried DCM. The flask was capped with a rubber septum and cooled to 0 °C. With stirring, methanesulfonyl chloride (5.0 g, 1.3 equiv) was added dropwise into the flask via syringe. The resulting mixture was stirred for 12 h at room temperature. After that, the mixture was extracted with DCM and washed with brine and water. The organic layer was dried with Na_2SO_4 and concentrated. The crude product was purified by flash column chromatography on silica gel using the eluent (DCM/hexane) to afford the product (7.0 g) as colorless liquid. Yield: 86%. ^{1}H NMR (500 MHz, Chloroform-*d*) δ 5.76 – 5.57 (m, 1H), 5.36 – 4.98 (m, 1H), 4.22 (t, *J* = 6.6 Hz, 2H), 3.00 (s, 3H), 2.42 (m, 1H), 2.19 (q, *J* = 10.5 Hz, 1H), 2.02 (m, 1H), 1.93 – 1.04 (m, 14H). ^{13}C NMR (101 MHz, Chloroform-*d*) δ 135.02, 129.80, 70.22, 37.35, 36.57, 36.03, 35.67, 29.59, 29.24, 26.90, 26.78, 25.79, 23.74.

5: In a 250 mL flask equipped with a septum and a magnetic stirrer under N2 atmosphere, N-methyl-trifluoroacetamide (3.4 g, 1 equiv.) was dissolved in anhydrous DMF (40 mL). The mixture was cooled to 0 °C, sodium hydride (0.68 g, 1.1 equiv) was slowly added, and the reaction mixture was stirred for 1 hour at room temperature. Then, **4** (6.0 g, 1 equiv) previously dissolved in anhydrous DMF (15 mL) was added dropwise via syringe and the resulting solution was heated at 70 °C for 12 hours. The reaction mixture was poured on water (100 mL) and extracted with EA (4x50 mL). The combined organic layers were dried over anhydrous Na_2SO_4, filtered and concentrated *in vacuo*. The crude product was purified by flash column chromatography on silica gel using the eluent (DCM/hexane) to afford the product (3.7 g) as colorless liquid. Yield: 50%. ^{1}H NMR (400 MHz, Chloroform-*d*) δ 5.76 - 5.57 (m, 1H), 5.36 – 4.98 (m, 1H), 3.48 – 3.31 (m, 2H), 3.15 – 2.95 (m, 3H), 2.50 – 2.35 (m, 1H), 2.25 – 2.10 (m, 1H), 2.10 – 1.95 (m, 1H), 1.84 – 0.84 (m, 14H). ^{13}C NMR (101 MHz, Chloroform-*d*) δ 135.04 (d, *J* = 23.7 Hz), 129.78 (d, *J* = 22.0 Hz), 117.97 (q, *J* = 320.0 Hz), 49.65 (d, *J* = 3.1 Hz), 49.45, 36.57, 36.27, 36.10, 35.71, 35.65, 29.60, 28.33, 26.92 (d, *J* = 1.6 Hz), 26.77, 26.55, 25.80 (d, *J* = 3.0 Hz), 24.83, 24.73.

6: Compound **5** (3.7 g, 1 equiv) was dissolved in MeOH (20 mL) and an aqueous solution of KOH (20 equiv, 20 mL of water) was added dropwise via a dropping funnel at 0 °C. The mixture was stirred at room temperature for 12 hours. Then, the MeOH was removed by vacuum, and the reaction mixture was poured on water (70 mL) and extracted with Et_2O (4 x 35 mL). The combined organic layers were dried over anhydrous Na_2SO_4, filtered and concentrated to give the expected product as a colorless liquid (2.35 g, 95%). ^{1}H NMR (400 MHz, Chloroform-*d*) δ 5.58 – 5.41 (m, 1H), 5.12 – 5.00 (m, 1H), 2.60 – 2.15 (m, 6H), 2.15 – 2.00 (m, 1H), 1.95 – 1.82 (m, 1H), 1.70 – 0.89 (m, 14H). ^{13}C NMR (101 MHz, Chloroform-*d*) δ 135.40, 129.24, 51.89, 36.58, 36.50, 36.11, 35.71, 29.77, 29.54, 26.83, 26.66, 25.76, 25.50.

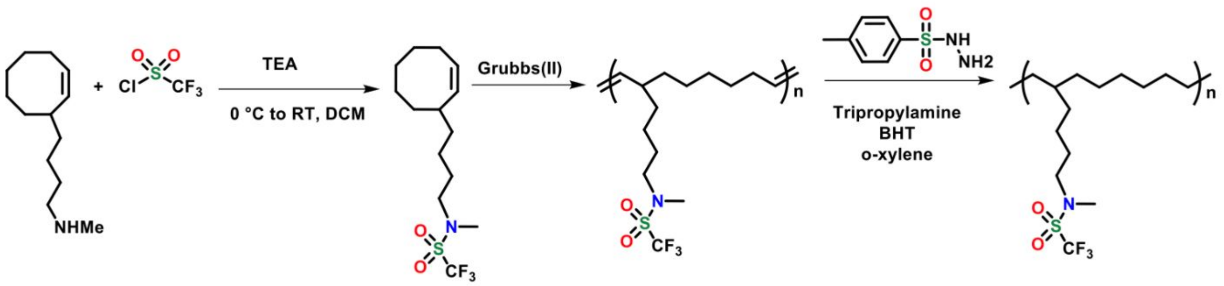

6 7 8 9

Figure S19: Reaction pathway for synthesis of P_CODC$_4$CF$_3$SA

7: To a 100 mL round-bottomed flask equipped with a magnetic stirring bar were added **6** (1.0 g, 1 equiv) and triethylamine (1.03 g, 2 equiv), followed by the addition of 20 mL freshly dried DCM. The flask was capped with a rubber septum and cooled to -78 °C. With stirring, trifluoromethanesulfonyl chloride (1.29 g, 1.5 equiv) in 10 mL DCM was added dropwise into the flask via syringe. The resulting mixture was allowed to warm and stirred for 12 h at room temperature. After that, the solid was filtered, and the filtrate was directly applied to flash column chromatography on silica gel using the eluent (DCM/hexane) to afford the product (1.10 g) as colorless liquid. Yield: 66%. ^{1}H NMR (400 MHz, Chloroform-*d*) δ 5.75 – 5.58 (m, 1H), 5.37 – 5.09 (m, 1H), 3.60 – 3.10 (m, 2H), 3.01 (s, 3H), 2.58 – 2.30 (m, 1H), 2.25 – 2.10 (m, 1H), 2.07 – 1.91 (m, 1H), 1.75 – 0.93 (m, 14H). ^{13}C NMR (101 MHz, Chloroform-*d*) δ 135.00, 129.81, 50.90, 36.58, 36.03, 35.66, 34.95, 29.61, 27.72, 26.92, 26.78, 25.80, 24.43

8: A 20 mL vial with a septum cap was charged with a stir bar, **7** (1.0 g, 1 equiv) and 5 mL dry DCM was added. The solution was bubbled with N_2 for for 15 min. Then Grubbs 2nd-generation catalyst (0.5 mol%) dissolved in 2 mL dried DCM was quickly injected via syringe. The reaction mixture was allowed to stir at room temperature over 5 h. ^{1}H-NMR was used to monitor the conversion of monomer. After full conversion, DCM was removed in vacuum to afford brown polymers. ^{1}H NMR (400 MHz, Chloroform-*d*) δ 5.45 – 5.25 (m, 1H), 5.12 – 4.90 (m, 1H), 3.50 – 3.10 (m, 2H), 3.00 (s, 3H), 2.23 – 1.78 (m, 3H), 1.68 – 1.03 (m, 14H). ^{13}C NMR (101 MHz, Chloroform-*d*) δ 134.34, 130.66, 120.26 (q, J = 323.9 Hz), 53.44, 50.91, 42.77, 35.63, 34.91, 34.80, 32.63, 29.72, 29.35, 27.11, 23.89.

9 (**P_CODC$_4$CF$_3$SA**): A mixture of polymer **8** (1.0 g, 1.0 equiv of olefin), *p*-toluenesulfonhydrazide (5.0 equiv), tripropylamine (5.0 equiv), a catalytic amount of BHT (ca. 10 mg), and o-xylene (50 mL) was refluxed for 8 h, and then allowed to cool to room temperature. The reaction mixture was poured into cold methanol. The precipitated polymer was isolated by decantation and purified by repeating reprecipitation using DCM/methanol system. The polymer was vacuum dried overnight to afford hydrogenated polymer products. Yield 48%. ^{1}H NMR (400 MHz, Chloroform-*d*) δ 3.13 – 3.00 (m, 2H), 2.73 (s, 3H), 2.72 (s, 6H), 1.55 – 1.40 (m, 2H), 1.30 – 1.07 (m, 19H). ^{13}C NMR (101 MHz, Chloroform-*d*) δ 120.27 (q, J = 324.0 Hz), 50.94, 37.38, 34.94, 33.61, 33.09, 30.24, 29.83, 28.11, 26.75, 23.33. ^{19}F NMR (376 MHz, Chloroform-*d*) δ -75.06. T_g = -29.0 °C.

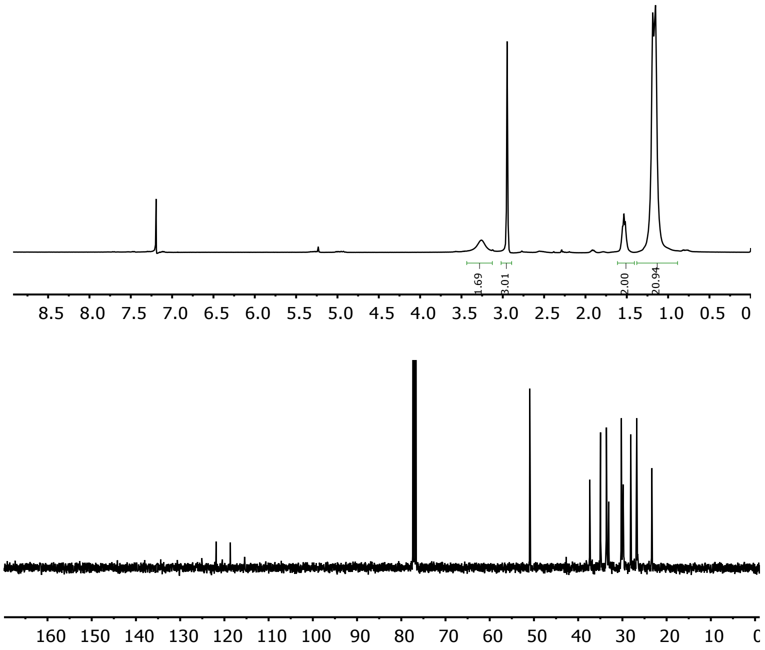

*Figure S20: ^{1}H (top) and ^{13}C (bottom) NMR spectra for **9**.*

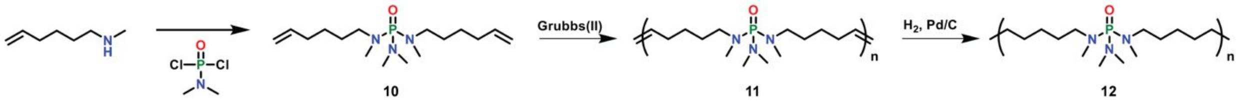

Figure S21: Reaction pathway for synthesis of P_C$_{10}$PA_MC

10: To a 100 mL round-bottomed flask equipped with a magnetic stirring bar were added *N*-methylhex-5-en-1-amine (0.70 g, 6.20 mmol), triethylamine (1.01 g, 10.0 mmol), and 20 mL freshly dried DCM. The mixture was cooled to -78 °C. With stirring, dimethylphosphoramidic dichloride (0.39 g, 2.41 mmol) in 3 mL DCM was added dropwise into the flask via syringe. The resulting mixture was allowed to warm and stirred for 12 h at room temperature. After that, the solid was filtered, and the filtrate was directly applied to flash column chromatography on silica gel using the eluent (DCM/methanol) to afford the product (0.71 g) as colorless liquid. Yield: 92%. ^{1}H NMR (400 MHz, Chloroform-*d*) δ 5.72 – 5.62 (m, 2H), 5.00 – 4.68 (m, 4H), 2.92 – 2.68 (m, 4H), 2.49 (d, J = 9.3 Hz, 6H), 2.47 (d, J = 9.3 Hz, 6H), 1.94 (q, J = 7.1 Hz, 4H), 1.41 (p, J = 7.3 Hz, 4H), 1.31 – 1.16 (m, 4H). ^{13}C NMR (101 MHz, Chloroform-*d*) δ 138.71, 114.55, 49.11 (d, J = 3.7 Hz), 36.95 (d, J = 3.8 Hz), 33.86 (d, J = 3.8 Hz), 33.58, 27.76, 26.20. ^{31}P NMR (162 MHz, Chloroform-*d*) δ 24.95 (dt, J = 18.4, 9.2 Hz).

11: A 8 mL vial was charged with a stir bar, monomer (0.3 g) and 0.6 mL dried DCM. The solution was bubbled with nitrogen for 15 min. Then Grubbs 2nd-generation catalyst (0.5 mol%) in 0.2 mL dried DCM was quickly injected via syringe. The reaction mixture was allowed to stir under nitrogen atmosphere for 0.5 h, then allowed to react at 50 °C under vacuum for 24 h. ^{1}H NMR spectroscopy was used to monitor the conversion of monomer. After full conversion, brown polymer **11** was collected. ^{1}H NMR (400 MHz, Chloroform-*d*) δ 5.38 – 5.27 (m, 2H), 2.94 – 2.69 (m, 4H), 2.60 – 2.48 (m, 12H), 2.00 – 1.85 (m, 4H), 1.55 – 1.40 (m, 4H), 1.35 – 1.15 (m, 4H). ^{31}P NMR (162 MHz, Chloroform-*d*) δ 25.13.

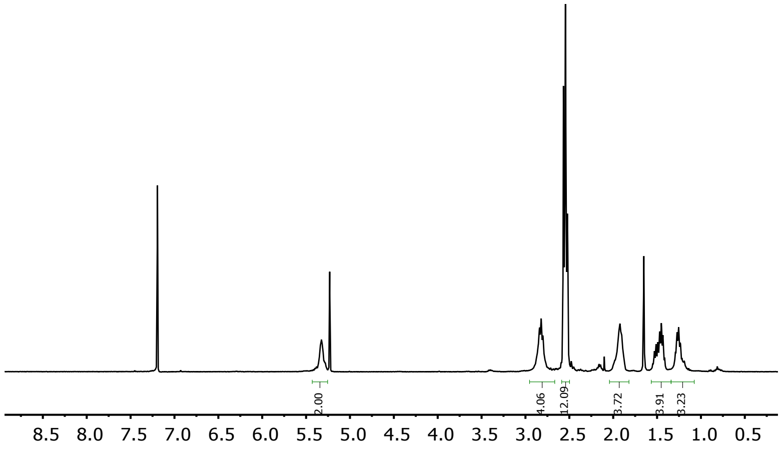

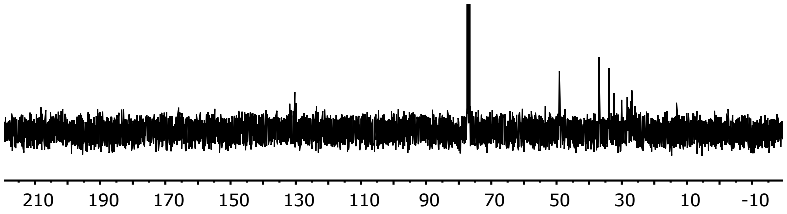

*Figure S22: ^{1}H (top) and ^{13}C (bottom) NMR spectra for **11**.*

12 (**P_C_{10}PA_MC**): A 50 mL round-bottle-flask with a septum cap was charged with a stir bar, polymer **11** (0.5 g), and 15 mL acetone. The solution was bubbled with nitrogen for 15 min. Then Pd/C (10% wt, 120 mg) was quickly added. The nitrogen was removed by vacuum, and a hydrogen balloon with a needle was quickly inserted to flask. The reaction mixture was stirred under hydrogen balloon for 0.5 h to 2 h, monitored by ^{1}H NMR. After full conversion, the reaction mixture was filtered through Celite, concentrated, and vacuum dried overnight to afford hydrogenated polymer products. ^{1}H NMR (400 MHz, Chloroform-*d*) δ 2.93 – 2.73 (m, 4H), 2.60 – 2.48 (m, 12H), 1.57 – 1.38 (m, 4H), 1.28 – 1.12 (s, 12H). ^{13}C NMR (101 MHz, Chloroform-*d*) δ 48.27, 35.95 (d, J = 3.8 Hz), 32.84 (d, J = 3.8 Hz), 28.63 (d, J = 4.9 Hz), 28.52 (d, J = 3.7 Hz), 27.34, 25.98 (d, J = 2.0 Hz). T_g = -42.8 °C.

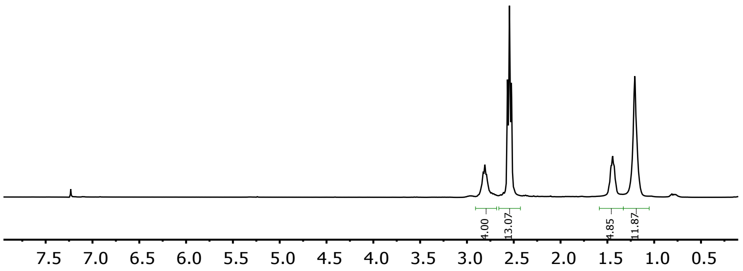

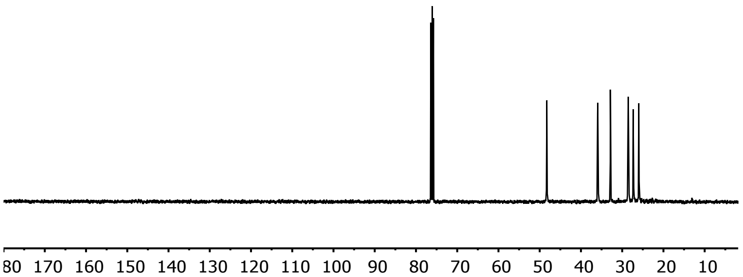

*Figure S23: ^{1}H (top) and ^{13}C (bottom) NMR spectra for **12**.*

Chemical structures of anions screened with PEO and PTMC

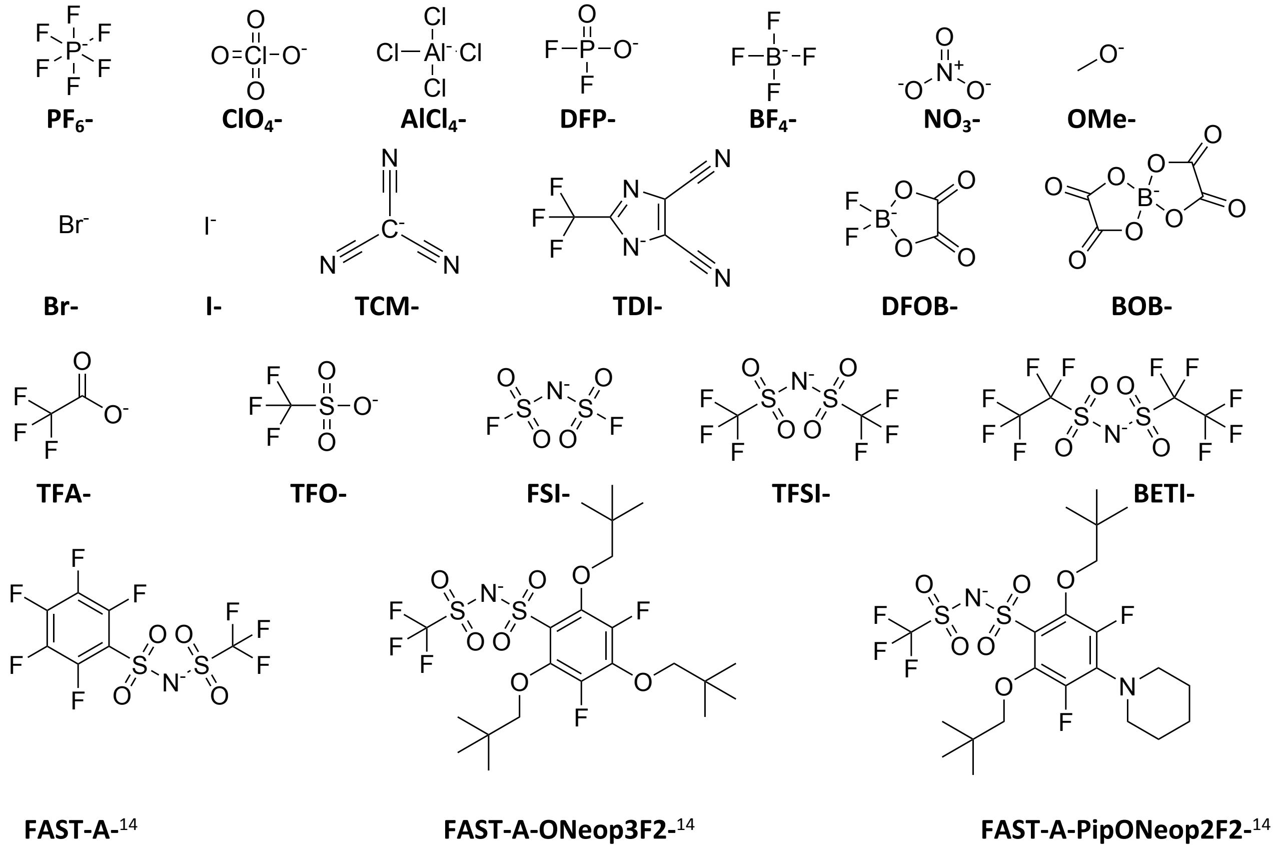

Figure S24: Chemical structures of anions used for screening of hypothetical SPEs. Anions were selected to represent a range of different anion chemistries.

Table S7: Salt concentrations reported in our database for anions screened with PEO and PTMC.

Polymer	Salt Anion	Concentration range (mol/kg)
	TFSI	0.05 - 15.2
	FSI	0.57 - 7.9
	TDI	0.03 - 5.7
	PF_6	0.23 - 11.4
	TFA	1.26 - 5.7
	TFO	0.11 - 11.4
	BOB	0.23 - 4.5
PEO	DFOB	0.76 - 2.8
	TCM	0.71 - 1.9
	BETI	0.45 - 5.7
	BF_4	0.45 - 11.4
	Br	0.51 - 204
	I	1.26 - 53
	$AlCl_4$	0.45 - 11.4
	ClO_4	0.0000101 - 7.6
	TFSI	0.47 - 4.9
	PF_6	0.1 - 4.1
PTMC	TFO	0.12 - 1.4
	BF_4	0.12 - 3.5
	ClO_4	0.13 - 4.9

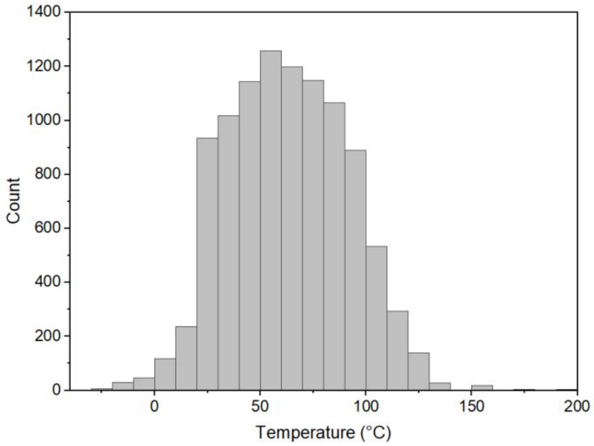

Figure S25: Histogram of temperatures at which ionic conductivity is reported generated from our database.

References

1. Hatakeyama-Sato, K., Tezuka, T., Umeki, M. & Oyaizu, K. AI-Assisted Exploration of Superionic Glass-Type Li+ Conductors with Aromatic Structures. *J Am Chem Soc* **142**, 3301–3305 (2020).
2. Yang, K. *et al.* Analyzing Learned Molecular Representations for Property Prediction. *J Chem Inf Model* **59**, 3370–3388 (2019).
3. Pesko, D. M. *et al.* Universal Relationship between Conductivity and Solvation-Site Connectivity in Ether-Based Polymer Electrolytes. *Macromolecules* **49**, 5244–5255 (2016).
4. Xue, Z., He, D. & Xie, X. Poly(ethylene oxide)-based electrolytes for lithium-ion batteries. *Journal of Materials Chemistry A* vol. 3 19218–19253 Preprint at https://doi.org/10.1039/c5ta03471j (2015).
5. Liu, R., Glover, K. P., Feasel, M. G. & Wallqvist, A. General Approach to Estimate Error Bars for Quantitative Structure–Activity Relationship Predictions of Molecular Activity. *J Chem Inf Model* **58**, 1561–1575 (2018).
6. Lascaud, S. *et al.* Phase Diagrams and Conductivity Behavior of Poly(ethylene oxide)-Molten Salt Rubbery Electrolytes. *Macromolecules* **27**, 7469–7477 (2002).
7. Matsumoto, K., Kakehashi, M., Ouchi, H., Yuasa, M. & Endo, T. Synthesis and Properties of Polycarbosilanes Having 5-Membered Cyclic Carbonate Groups as Solid Polymer Electrolytes. *Macromolecules* **49**, 9441–9448 (2016).
8. Silva, M. M., Barros, S. C., Smith, M. J. & MacCallum, J. R. Characterization of solid polymer electrolytes based on poly(trimethylenecarbonate) and lithium tetrafluoroborate. *Electrochim Acta* **49**, 1887–1891 (2004).
9. Timachova, K., Watanabe, H. & Balsara, N. P. Effect of molecular weight and salt concentration on ion transport and the transference number in polymer electrolytes. *Macromolecules* **48**, 7882–7888 (2015).
10. Teran, A. A., Tang, M. H., Mullin, S. A. & Balsara, N. P. Effect of molecular weight on conductivity of polymer electrolytes. *Solid State Ion* **203**, 18–21 (2011).
11. Timachova, K., Watanabe, H. & Balsara, N. P. Effect of molecular weight and salt concentration on ion transport and the transference number in polymer electrolytes. *Macromolecules* **48**, 7882–7888 (2015).
12. Diederichsen, K. M., McShane, E. J. & McCloskey, B. D. Promising Routes to a High Li+ Transference Number Electrolyte for Lithium Ion Batteries. *ACS Energy Lett* **2**, 2563–2575 (2017).
13. Gorecki, W., Jeannin, M., Belorizky, E., Roux, C. & Armand, M. Physical properties of solid polymer electrolyte PEO(LiTFSI) complexes. *Journal of Physics: Condensed Matter* **7**, 6823 (1995).
14. Huang, M. *et al.* Fluorinated Aryl Sulfonimide Tagged (FAST) salts: modular synthesis and structure–property relationships for battery applications. *Energy Environ Sci* **11**, 1326–1334 (2018).
